# Recombinant vesicular stomatitis vaccine against Nipah virus has a favorable safety profile: Model for assessment of live vaccines with neurotropic potential

**DOI:** 10.1371/journal.ppat.1010658

**Published:** 2022-06-27

**Authors:** Thomas P. Monath, Richard Nichols, Lynda Tussey, Kelly Scappaticci, Thaddeus G. Pullano, Mary D. Whiteman, Nikos Vasilakis, Shannan L. Rossi, Rafael Kroon Campos, Sasha R. Azar, Heidi M. Spratt, Brent L. Seaton, W. Tad Archambault, Yanina V. Costecalde, Evan H. Moore, Roger J. Hawks, Joan Fusco

**Affiliations:** 1 Public Health Vaccines LLC, Cambridge, Massachusetts, United States of America; 2 Crozet BioPharma Inc., Lexington, Massachusetts, United States of America; 3 BioReliance Corporation, Rockville, Maryland, United States of America; 4 Department of Pathology, University of Texas Medical Branch, Galveston, Texas, United States of America; 5 Sealy Center for Vector-Borne and Zoonotic Diseases, University of Texas Medical Branch, Galveston, Texas, United States of America; 6 Department of Preventive Medicine and Population Health, University of Texas Medical Branch, Galveston, Texas, United States of America; 7 Q2 Solutions, San Juan Capistrano, California, United States of America; 8 VirtuStat, North Wales Pennsylvania, United States of America; 9 AmplifyBio, West Jefferson, Ohio, United States of America; 10 Battelle Memorial Institute, West Jefferson, Ohio, United States of America; University of Pittsburgh, UNITED STATES

## Abstract

Nipah virus (NiV) disease is a bat-borne zoonosis responsible for outbreaks with high lethality and is a priority for vaccine development. With funding from the Coalition of Epidemic Preparedness Innovations (CEPI), we are developing a chimeric vaccine (PHV02) composed of recombinant vesicular stomatitis virus (VSV) expressing the envelope glycoproteins of both Ebola virus (EBOV) and NiV. The EBOV glycoprotein (GP) mediates fusion and viral entry and the NiV attachment glycoprotein (G) is a ligand for cell receptors, and stimulates neutralizing antibody, the putative mediator of protection against NiV. PHV02 is identical in construction to the registered Ebola vaccine (Ervebo) with the addition of the NiV G gene. NiV ephrin B2 and B3 receptors are expressed on neural cells and the wild-type NiV is neurotropic and causes encephalitis in affected patients. It was therefore important to assess whether the NiV G alters tropism of the rVSV vector and serves as a virulence factor. PHV02 was fully attenuated in adult hamsters inoculated by the intramuscular (IM) route, whereas parental wild-type VSV was 100% lethal. Two rodent models (mice, hamsters) were infected by the intracerebral (IC) route with graded doses of PHV02. Comparator active controls in various experiments included rVSV-EBOV (representative of Ebola vaccine) and yellow fever (YF) 17DD commercial vaccine. These studies showed PHV02 to be more neurovirulent than both rVSV-EBOV and YF 17DD in infant animals. PHV02 was lethal for adult hamsters inoculated IC but not for adult mice. In contrast YF 17DD retained virulence for adult mice inoculated IC but was not virulent for adult hamsters. Because of the inconsistency of neurovirulence patterns in the rodent models, a monkey neurovirulence test (MNVT) was performed, using YF 17DD as the active comparator because it has a well-established profile of quantifiable microscopic changes in brain centers and a known reporting rate of neurotropic adverse events in humans. In the MNVT PHV02 was significantly less neurovirulent than the YF 17DD vaccine reference control, indicating that the vaccine will have an acceptable safety profile for humans. The findings are important because they illustrate the complexities of phenotypic assessment of novel viral vectors with tissue tropisms determined by transgenic proteins, and because it is unprecedented to use a heterologous comparator virus (YF vaccine) in a regulatory-enabling study. This approach may have value in future studies of other novel viral vectors.

## Author summary

Nipah virus (NiV) disease is a highly lethal bat-borne virus with epidemic potential causing inflammation of the brain and a severe respiratory syndrome and is a high priority for vaccine development. We developed a novel single-dose vaccine that protects animals against disease and death caused by NiV and have started clinical trials. The vaccine is a live, recombinant vesicular stomatitis virus (VSV) vector identical to the recently approved Ebola vaccine (Ervebo) but also expressing the NiV G protein responsible for attachment of the virus to cell receptors. Vaccination results in antibodies to the G protein that block entry of the virus into cells. Since addition of the NiV receptor-binding G protein to a live virus could potentially target it to receptors on brain cells, extensive safety tests for neurovirulence were required involving direct inoculation of the vaccine virus into brains of different animal models. We showed that the vaccine candidate was significantly less neurovirulent in non-human primates than an unrelated approved live viral vaccine against yellow fever which has a long record of safe use and a known incidence of rare neurological adverse events. The use of an unrelated vaccine as a comparator is unprecedented in regulatory science and provides a novel approach to safety testing that may be applicable to other vaccines.

## Introduction

This paper describes a novel live, attenuated vaccine against NIV, an important human pathogen, and the general problem of assessing safety of recombinant vectors expressing a foreign gene that may alter tropism and pathogenesis.

Nipah virus is a neurotropic, bat-borne Henipavirus (family *Paramyxoviridae*) responsible for human disease outbreaks in Southeast Asia and the western Pacific, characterized by encephalitis, pneumonitis, vasculitis, a high case-fatality rate, neurologic sequelae, and potential recrudescence in survivors [1]. It is a zoonotic infection transmitted by bats, fomites or food and drink contaminated with bat secretions, infected intermediate hosts (e.g., swine), or direct contact with infected humans [2]. Vaccines for prevention of Nipah virus disease are a high priority of the World Health Organization (WHO) and CEPI.

**Fig 1:**
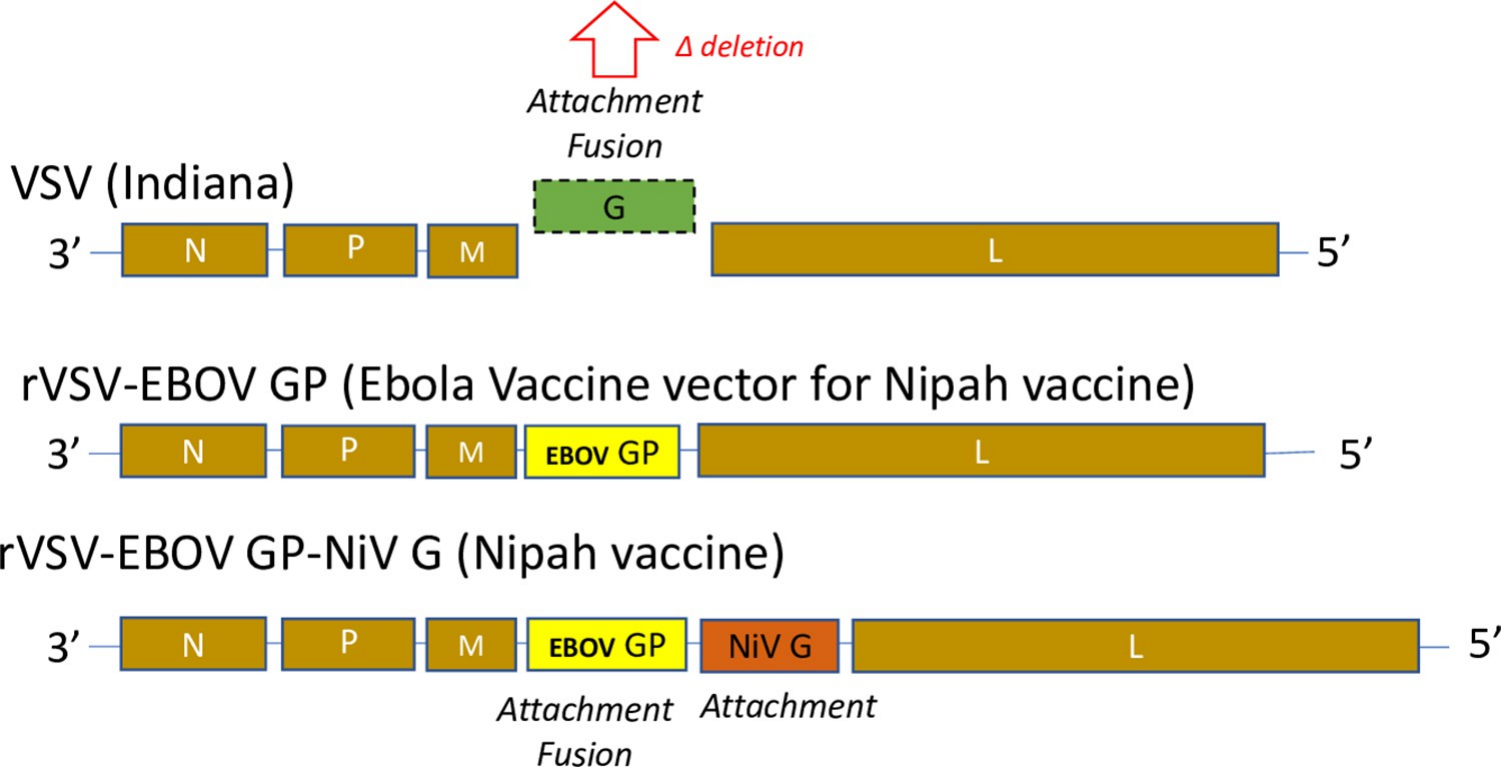
Schematic of genome organization of wild-type VSV (top), rVSV-EBOV (Ebola) vector (center) showing the substitution of the EBOV GP gene for the glycoprotein (G) gene and principal virulence factor of wild-type VSV, and rVSV-Nipah (bottom) showing the addition of NiV G encoding the NiV attachment glycoprotein downstream of the EBOV GP.

A recombinant vector vaccine candidate was engineered by the Feldmann Lab, Laboratory of Virology, Division of Intramural Research, NIAID, NIH which also showed that it raised protective immunity after a single dose in animal models, including nonhuman primates [3,4]. The vaccine is a replication-competent recombinant VSV (Indiana) with the VSV envelope glycoprotein (G) gene deleted (VSVΔG) and replaced with envelope glycoprotein genes of two heterologous negative-sense single-strand RNA viruses: EBOV GP and NiV G (**Fig 1**). PHV02 is therefore a true chimera (three-headed creature, Greek mythology), a virus having three different parts each of which contributes to its biological phenotype. While safety assessment of the final drug product to be clinically tested is the ultimate regulatory desideration, dissection of the role of the independent genetic components is important to understanding its pathogenesis. Many of the considerations for safety assessments of the chimeric vector are generalizable and may help to inform other vaccine development efforts.

There are now several vaccines approved for human use based on live, replication competent recombinant viral vectors, including rVSV-EBOV (Ervebo) [5,6], recombinant yellow fever (YF) 17D-dengue (Dengvaxia) [7], and YF 17D-Japanese encephalitis (ImoJev) [8], and development of other viral vectors remains an area of wide research interest [9–20]. In all cases, a challenge for vaccine development is to confirm safety of a novel genetically modified organism (GMO) having virulence, tropism, and persistence characteristics derived from both the parental virus and the incorporated transgene(s).

Live, attenuated vaccines are some of the most effective vaccines because they expand the antigenic mass, require relatively low doses and often a single administration, activate innate immune genes that provoke adaptive responses, elicit rapid-onset and durable immunity, and present antigens via the endogenous pathway for strong CD8+ responses. However, the replicative capacity and ability to spread creates safety concerns, especially in individuals unable to mount innate or adaptive responses necessary to clear the vaccine infection, or in whom the virus is able to invade and damage the developing fetus or a susceptible organ from which it is normally excluded, in particular the central nervous system (CNS). For these reasons, safety assessments of new live vaccines involve toxicity tests in animals, biodistribution studies, and tests of neurovirulence employing direct IC inoculation. Neuroinvasiveness (the ability of a virus to invade the central nervous system after peripheral inoculation) is assessed as a component of biodistribution and toxicology studies, whereas neurovirulence is assessed by direct IC inoculation and by-passing the blood-brain barrier and reveals the worst-case potential for viral injury.

Tests for neurovirulence of novel live vaccines are required on a case-by-case basis [21,22]. Neurovirulence tests, and in particular the MNVT, are mandatory when the parental virus or the transgene(s) donor (or both) are inherently neurotropic. This was illustrated in the case of a chimeric tick-borne encephalitis (TBE)/dengue 4 vector, in which the TBE transgene conferred neurovirulence on the otherwise apathogenic dengue 4 parent [23]. The vaccine under consideration here (rVSV-Nipah) expresses the G protein of NiV which is responsible for attachment to ephrin B2 and ephrin B3 receptors to initiate cell infection [24] and could potentially increase neurovirulence. Ephrin receptors are abundantly expressed on neural cells, and human NiV disease is characterized by neuroinvasion and encephalitis [25]. Therefore, prior to human trials, the rVSV-Nipah vaccine must be evaluated for its ability to invade, infect, and cause damage to the CNS using test methods acceptable to regulatory authorities.

The rVSV-Nipah vector was constructed to include both the EBOV GP and NiV G proteins. Thus, it is a partial replica of the rVSV-EBOV (Ervebo, Merck & Co.) vaccine, which is approved by the US Food & Drug Administration (FDA), the European Medicines Agency and WHO, with the addition of the gene encoding the NiV G protein. The EBOV GP, which replaces the deleted G protein of VSV, the principal neurovirulence VSV gene, results in marked attenuation [8,26]. Additionally, EBOV GP substitutes for VSV G in mediating fusion of the recombinant virus envelope with the cell membrane required for entry of viral RNA into the cytoplasm. The NiV G protein does not subserve fusion and without EBOV GP, the vector virus would not enter cells. The use of an attenuated, authorized vaccine (rVSV-EBOV) as a vector simplifies phenotypic evaluation and toxicity testing, since head-to-head comparison of it and the investigational product which differs only in expressing NiV G, enables dissection of the role of the latter protein in virulence. We therefore compared the neurovirulence for mice of IC inoculation of the rVSV-Nipah vector with that of rVSV-EBOV (lacking Nipah G).

Here we report the results of toxicology studies on the rVSV-Nipah vaccine, including a MNVT, which is required for live viral vaccines derived from a neurotropic progenitor [27]. In addition to use of rVSV-EBOV as a comparator, we use YF vaccine as an active control in neurovirulence testing, since it has residual neurotropism against which the new vaccine can be benchmarked, a known reporting rate of neurotropic adverse events in humans, and a quantifiable pattern of pathological changes in the brain of monkeys after IC inoculation [28]. The result of these investigations showed that the rVSV-Nipah vaccine candidate had a favorable safety profile compared to YF vaccine, with virtual absence of neurovirulence in the MNVT, suggesting that the vaccine candidate will be as safe or safer than YF, a live, attenuated vaccine in wide human use. The use of a heterologous virus in regulatory-enabling toxicology studies is, to our knowledge, unprecedented.

## Results

**Fig 2:**
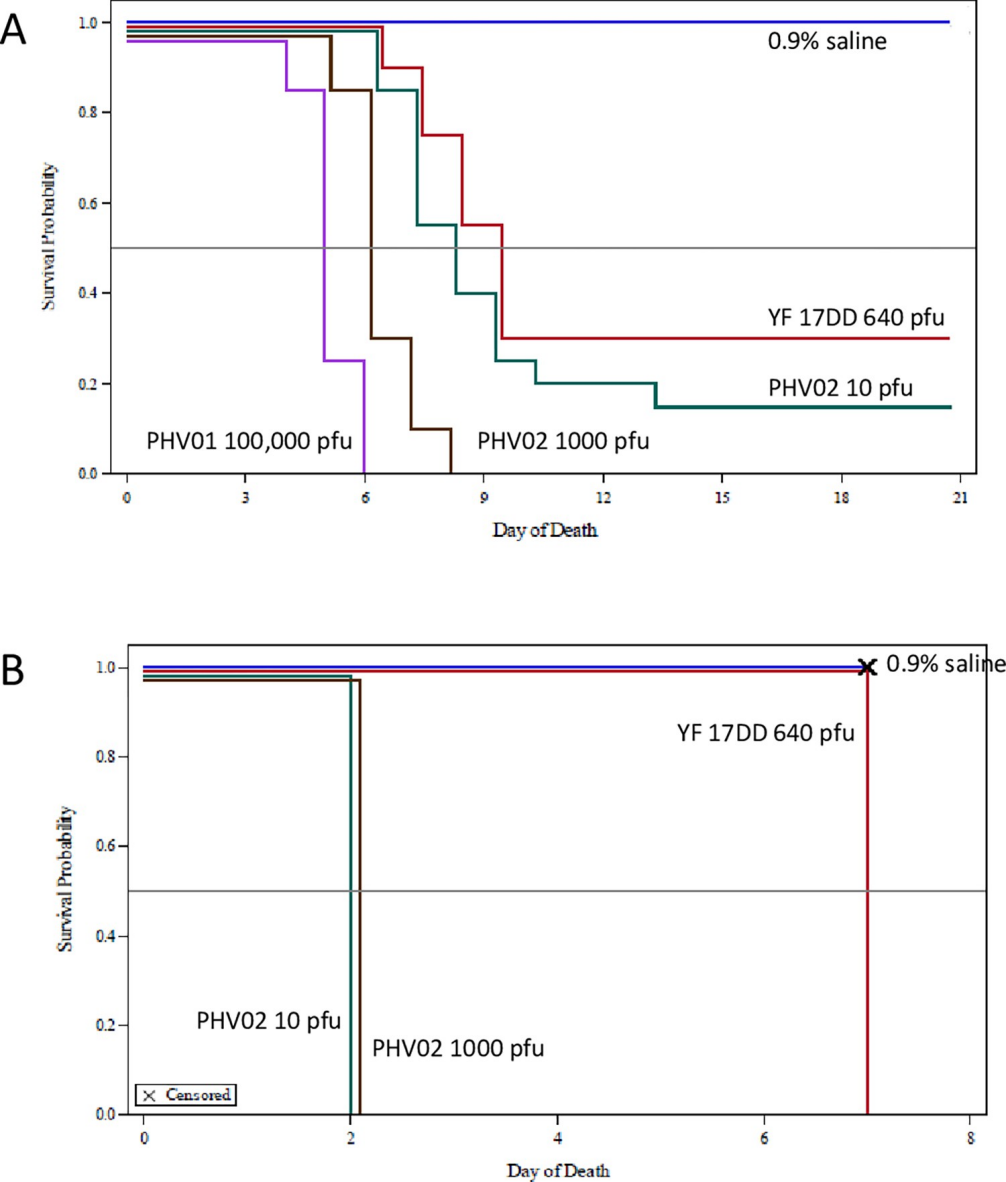
Survival distribution, infant mice and hamsters inoculated by the IC route. **A. 8-day-old ICR mice.** Twenty μL of PHV02 at graded doses or YF 1DD vaccine were inoculated into 2 litters (10 suckling pups/litter). The doses injected in 20 μL were 100,000, 1000, or 10 pfu for PHV02 groups and 640 pfu for YF 17DD. Animals were observed for 21 consecutive days and clinical signs and deaths recorded. All mice died in the intermediate and high dose groups, 85% died in the low dose (10 pfu) group and 70% died in the reference group receiving 640 pfu of YF17DD. **B. 8-day-old Golden hamsters.** Twenty μL of PHV02 at graded doses or YF 17DD were inoculated into 2 litters (8-10 suckling pups/litter). The doses injected in 20 μL were 1000 or 10 pfu for PHV02 groups and 640 pfu for YF 17DD. All infant hamsters died, but the YF 17DD animals had a statistically longer survival time (see text). Since all animals in the test article groups died by Day 7, the study was terminated at that point.

**Fig 3:**
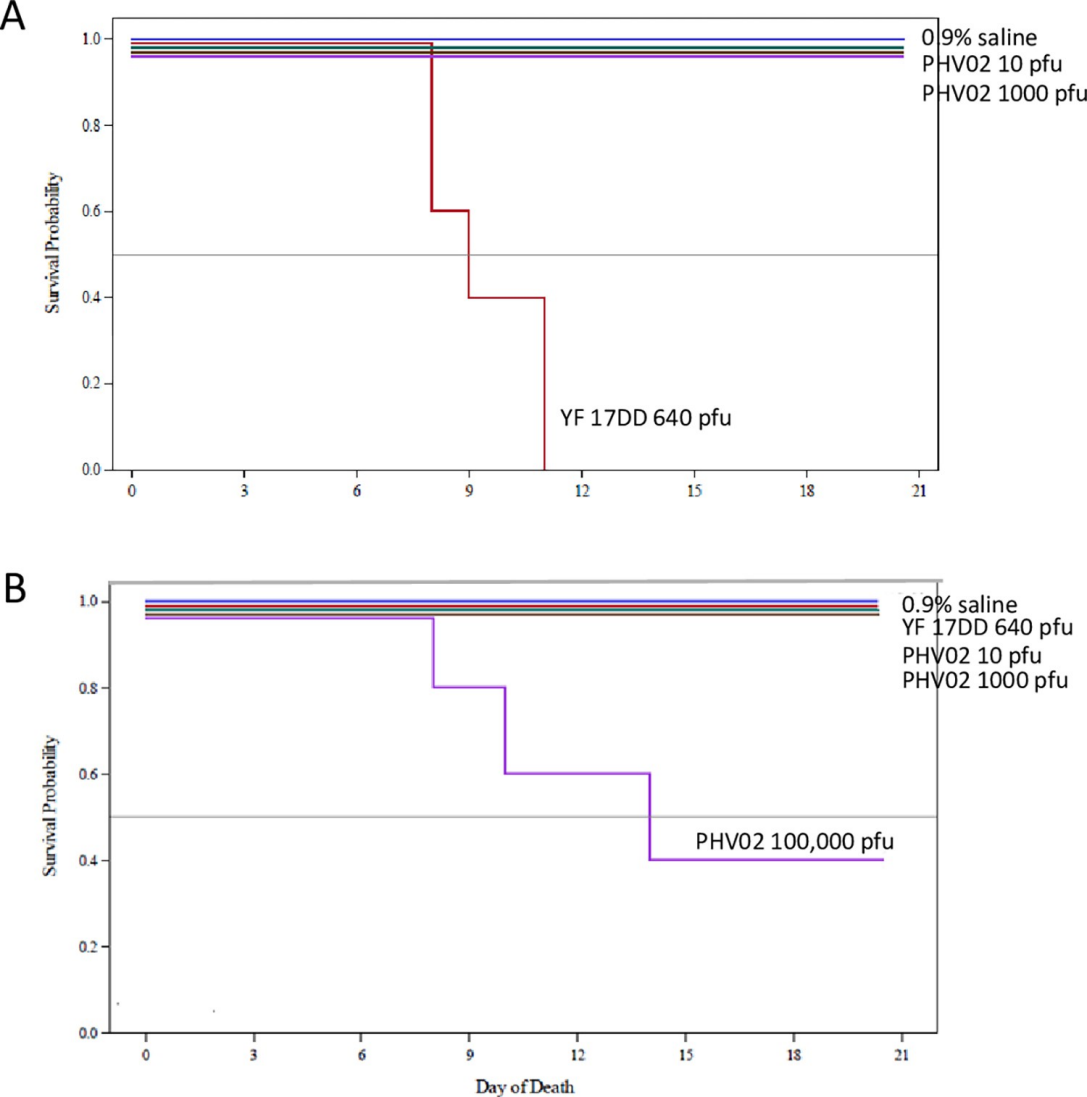
Survival distribution, 6- to 8-week-old ICR mice and Golden hamsters inoculated by the IC route. **6- to 8-week-old ICR mice** Twenty μL of PHV02 at graded doses or YF 17DD were inoculated IC into groups of 5 mice. The doses injected in 20 μL were 100,000, 1000, or 10 pfu for PHV02 groups and 640 pfu for YF 17DD. **6- to 8-week-old Golden hamsters.** Twenty μL of PHV02 or YF 17DD were inoculated IC into groups of 5 hamsters. The doses injected in 20 μL were 100,000, 1000, or 10 pfu for PHV02 groups and 640 pfu for YF 17DD. Animals were observed for 21 consecutive days and clinical signs and deaths recorded. Hamsters inoculated with 100,000 pfu of PHV02 virus had a 60% mortality ratio for an IC LD50 of 46,800 pfu. In contrast to adult mice which are susceptible to lethal IC infection with YF 17DD **Fig 3A**), adult hamsters showed no illness or deaths.

**Fig 4:**
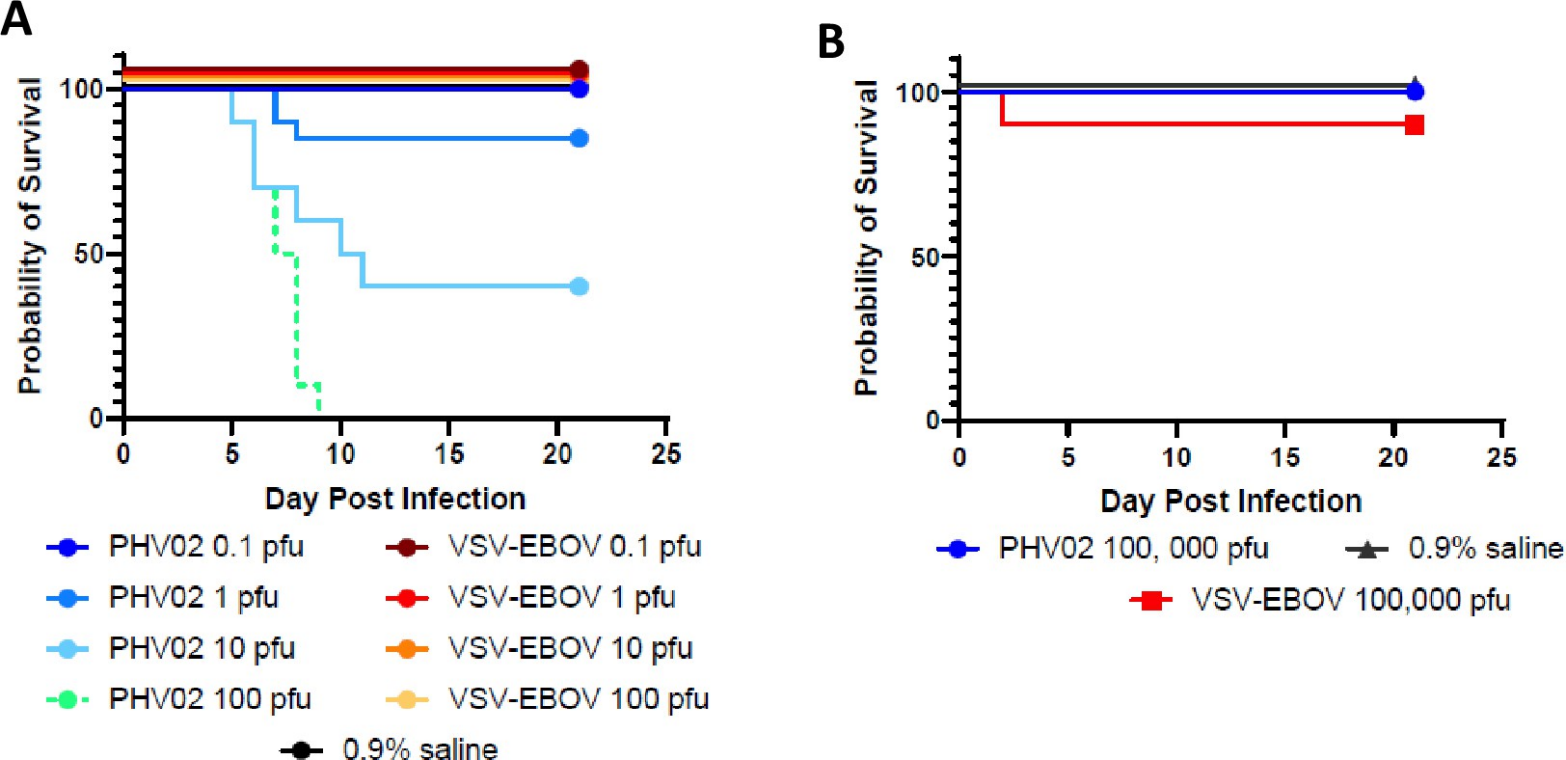
Survival distribution of infant and adult Swiss-Webster mice inoculated IC with rVSV-Nipah or rVSV-EBOV. **A. 8-day-old mice (10 animals/group) inoculated IC with 0.1, 1, 10 or 100 pfu of PHV02 (VSV-Nipah virus), rVSV-EBOV, or 0.9% saline**. rVSV-EBOV was avirulent at all dose levels. The IC LD50 for PHV02 is 5.3 pfu, and the LD50 of rVSV-EBOV is >100 pfu. **B. 6- to 8-week-old Swiss-Webster mice (10 animals/group) inoculated IC with 100,000 pfu of VSV-Nipah virus, rVSV-EBOV, or 0.9% saline**.

Two studies in rodents provided preliminary data on neurotoxicity of the PHV02 vaccine candidate. Infant and adult mice are widely used to determine neurovirulence of viruses, including rVSV viruses [29], YF 17D [30], and other viruses. Hamsters have been studied as a model for rVSV viruses, as well as wild-type Nipah [3] and YF viruses [31] and were therefore included for comparative analysis. The results showed that the PHV02 vaccine candidate was neurovirulent for infant mice and hamsters, and partially neurovirulent for adult hamsters but with a high median lethal dose (**Fig 2**). In adult mice, the PHV02 vaccine candidate was nonpathogenic whereas the YF vaccine (a commercial lot of YF 17DD vaccine) killed all animals (**Fig 3**). When compared to the Ebola vaccine (rVSV-EBOV) in infant mice and hamsters, PHV02 had a statistically higher mortality and shorter survival distribution (**Fig 4**). This indicated that the NiV G protein expressed by PHV02 imparted a higher degree of neurovirulence for infant animals. However, there were mixed results in the adult animals indicating both higher neurovirulence or greater attenuation compared to the approved YF 17DD vaccine depending on the rodent species and age. These mixed results warranted further evaluation in the MNVT, considered the gold-standard test [21,22,32].

### Pilot neurovirulence test in mice and hamsters of PHV02 vs. YF 17DD vaccine

Graded doses of PHV02 [10, 1000, 100,000 plaque forming units (pfu)] or 640 pfu of YF 17DD were inoculated by the IC route into ICR mice and Golden hamsters 2 days, 8 days or 6-8 weeks of age. The dose of YF 17DD employed was the highest dose possible (reconstituted lyophilized commercial vaccine without further dilution) and approximated the intermediate dose of the PHV02 test article. Negative controls were inoculated with 0.9% saline.

PHV02 and YF 17DD reference control (but not 0.9% saline) were lethal for 2 and 8-day old mice, and therefore results for more susceptible 2-day old animals are not presented. In 8-day old mice inoculated IC, PHV02 was 100% lethal at the 100,000 and 1000 pfu dose levels and 85% at the 10 pfu dose level (**Fig 2A**). The dose response for mortality was close to, but not statistically significant (p=0.0666, Cochran-Armitage test). However, the survival distribution among the 3 PHV02 dose groups showed a significant dose response (p < 0.0001, Cox PH model) for the log-concentration. The combined PHV02 mortality ratio (95%) was significantly higher than the 70% mortality rate in the YF 17DD group (p = 0.0063, Fisher’s exact test). The survival of 30% of 8 day-old mice inoculated IC with YF 17DD was unexpected, since all adult animals succumbed (see below). At closely matched dose levels of 1000 pfu of PHV02 and 640 pfu of YF 17DD, the survival distributions were significantly different (p<0.0001, log-rank test), showing an average survival time of ~7 days for PHV02 and ~9 days for YF 17DD. In infant mice, the higher mortality ratio and shorter survival distribution indicated that PHV02 was more neurovirulent than the YF 17DD reference control. This conclusion, was, however, not borne out in adult mice, where YF 17DD was significantly more lethal, as shown below.

All 2- and 8-day-old hamsters died when inoculated with 10 or 1000 pfu of PHV02 or with 640 pfu YF 17DD inoculated (8-day-old data shown in **Fig 2B**). The 100,000 pfu dose was not given to the 8-day-old animals since all 2-day-old animals had succumbed at a dose of ≥1000 pfu. Despite uniform lethality, a significant difference was found in survival distribution between the PHV02 groups (where all animals died on Day 2) and YF 17DD, where all animals died on Day 7 (p < 0.0001, log-rank test). Based on survival time, PHV02 virus is more neurovirulent for hamsters than for mice. This conclusion was supported by results in adult animals, as described below.

### Neurovirulence for adult mice and hamsters vs. YF 17DD

In contrast to infant mice, 6- to 8-week-old mice inoculated IC with graded doses (10, 1000,

100,000 pfu) of PHV02 showed no signs of illness and survived for 21 days (**Fig 3A**). In contrast, YF 17DD was 100% lethal for adult mice, with a mean survival time of 9 days. Neurovirulence of YF vaccine for adult mice is a well-known feature [33]. In this experiment, mice were inoculated with 640 pfu of the YF 17DD vaccine, which has an expected 50% lethal dose (LD50) of approximately 1 pfu [34]. PHV02 was not neurovirulent for adult mice and thus had an LD50 >100,000 pfu.

A different picture was observed in adult hamsters. Hamsters inoculated IC with the highest dose (100,000 pfu) of PHV02 virus had a 60% mortality ratio (**Fig 3B**). The IC LD50 for adult hamsters was 46,800 pfu. Hamsters inoculated with lower doses (10 or 1000 pfu) of PHV02 and those receiving YF 17DD or 0.9% saline showed no illness or deaths. The PHV02 dose group mortality ratios differed significantly (p = 0.0220, Cochran-Armitage test).

In conclusion, PHV02 virus was highly lethal for infant mice and hamsters and retained a degree of neurovirulence for adult hamsters, albeit with a high LD50 (46,800 pfu). In contrast to adult mice which remain susceptible to YF 17DD, adult hamsters show age-related resistance to YF 17DD virus inoculated by the IC route. Overall, the results in mice and hamsters showed PHV02 to have residual neurovirulence; the comparison of PHV02 with a human live vaccine, YF 17DD, yielded discordant results, with higher virulence of PHV02 for infant mice and hamsters and for adult hamsters, but attenuation in adult mice.

### Toxicity of PHV02 by peripheral Inoculation and attenuation vs. wild-type VSV

Since adult hamsters developed lethal infection after IC inoculation of PHV02, two studies were performed to determine susceptibility to peripheral infection. In a pilot study, groups of ten 8-week-old hamsters were inoculated by intramuscular (IM) injection (0.25 mL) with either PHV02 (1.06 x 10^7^ /animal), wild-type VSV Indiana (1.9 x 10^7^ pfu/animal) or 0.9% saline. Blood was collected via the retro-orbital sinus before and on 1, 3, 7 and 14 days after injection of the test articles. Animals were observed for clinical signs twice daily and weighed weekly. Complete and differential white cell counts and viremia measurements by qRT-PCR were performed on blood samples, and necropsies were performed on half of the animals on days 7 and 21 post inoculation. Since this study will be the subject of a separate report, only top-line data relevant to safety are provided here. None of the animals receiving PHV02 or 0.9% saline showed any signs of illness and most gained weight, whereas all 10 hamsters inoculated with wild-type VSV became ill including neurological signs and had high clinical scores starting 2 days after inoculation and culminating with humane euthanasia by day 6. Body weight changes, clinical scores, and survival distributions are provided in **S1 Fig. Toxicity of PHV02 compared to wild-type VSV (**Panels A-C). There were also significant changes in white cell counts in the wild-type VSV controls on days 4-6 (neutrophilia, monocytemia and lymphopenia) but not in the PHV02 or saline groups. Viremia levels were significantly higher than in the PHV02 treatment group on day 1 (p=0.027, *t* test 2-tailed) as shown in **S1 Fig. Toxicity of PHV02 compared to wild-type VSV** (Panel D). Viremia levels for VSV animals were higher on day 3 post inoculation, but differences did not reach statistical significance. The data indicate that PHV02 inoculated by the IM route produced active infection and viremia levels of 5-6 log10 copies/mL in hamsters without causing overt signs of illness. Histopathologic evaluation showed pneumonitis and peri-portal inflammation in the VSV treatment group whereas the PHV02 treated animals had minimal focal changes. There were no histopathologic changes in brain or spinal cord in any treatment groups; their absence in the VSV group (which had neurological signs of illness) probably reflects the hyperacute nature of the infection. This study demonstrated that while replication competent in vivo, PHV02 was highly attenuated compared to its wild-type VSV progenitor.

In a second formal toxicology study, groups of forty 8-week-old hamsters (20 males, 20 females) were inoculated (Day 1) by the IM route with PHV02 (Toxicology lot, 3.04 x 10^7^ pfu/animal), the approximated full human dose, or 0.9% saline. Based on the study of IC inoculation described earlier the dose administered was 650 adult hamster IC LD50. Half of the animals were sacrificed on Day 15 and the remainder on Day 29. None of the hamsters showed clinical abnormalities and all survived to the study endpoint. There were no injection site abnormalities, body temperature elevations, or test-article related changes in weight, hematology, coagulation, and clinical chemistry tests. On complete histopathological evaluation as specified by WHO [27], there were no changes in organ weights and few microscopic findings of low frequency, disparate character, and low severity grades. The inconsistent nature of these findings and their presence in both treated and control groups did not indicate a relationship to PHV02. All animals treated with PHV02 and tested on day 15 or 29 developed neutralizing antibodies, whereas all 0.9% saline controls were negative (**S2 Fig. Neutralizing antibody titers against wild-type Nipah virus, toxicology study**). On Day 15 the geometric mean titer was 1766 (95% CI 1023, 3050) and significantly increased by Day 29 [GMT 4721 (95% CI 3700, 6023), p=0.0402 (*t* test with Welsh’s correction]. Overall, it was concluded that IM inoculation of a high dose of PHV02 in a highly susceptible animal host species, resulted in a robust immune response with no clinical or pathological toxicities. These results were filed to and enabled the Investigational New Drug (IND) application for clinical trials.

### Comparative neurovirulence of PHV02 and rVSV-EBOV

A comparison of PHV02 and rVSV-EBOV was carried out in 8-day-old infant mice to define the role of the Nipah G protein in neurovirulence, to determine the median lethal dose, and to confirm attenuation in adult animals. PHV02 was 100% lethal in infant mice at the highest (100 pfu) dose level, 60% and 15% lethal at 10 and 1 pfu dose, respectively, and non-lethal at the 0.1 pfu dose level (**Fig 4A**). Time to death was dose-related, with an average survival time of 7.1 days at a dose of 100 pfu. The infant mouse IC LD50 of PHV02 was 5.3 pfu. No mortality was observed for the rVSV-EBOV comparator indicating an infant mouse IC LD50 of >100 pfu **(Fig 4A**). Infant mice inoculated with PHV02 10 or 100 pfu died or failed to gain weight, whereas those inoculated with lower doses or with rVSV-EBOV gained weight (**S3 Fig. Body weights, 8-day-old Swiss-Webster mice by day after IC inoculation of graded doses of rVSV-Nipah (PHV02) or rVSV-EBOV).** A subset of 5 mice was euthanized 5 days after inoculation for determination of viral loads in brain and spleen. Viral loads of PHV02 were dose dependent, but a dose effect was less evident for rVSV-EBOV (**S4 Fig. Viral load determined by qRT-PCR in brain and spleen tissues from Swiss-Webster mice**). Viral loads in the PHV02 100 pfu dose group were >2 logs higher than in the lower dose groups. The 100 pfu dose group was tested on Day 5 at onset of morbidity (**Fig 4A**). Interestingly, viral loads in the rVSV-EBOV groups, which survived the infection were similar to those in the PHV02 10 pfu group which experienced 60% lethal infection (**S4 Fig. Viral load determined by qRT-PCR in brain and spleen tissues from Swiss-Webster mice**). In contrast to infant mice, 5/5 (100%) 6- to 8-week-old mice inoculated IC with 100,000 pfu of PHV02, and 4/5 (80%) inoculated with rVSV-EBOV showed no signs of illness and survived for 21 days (**Fig 4B)**. The one early death in the rVSV-EBOV group (day 2 after inoculation) was probably non-specific since infant mice inoculated IC with the virus showed no mortality. No cause for death was determined. Viral loads on Day 5 in brain and spleen were similar across treatment groups and lower in adult than in infant mice **(S4 Fig. Viral load determined by qRT-PCR in brain and spleen tissues from Swiss-Webster mice**). The study confirmed that PHV02 was more neurovirulent than the rVSV-EBOV vaccine in a sensitive infant mouse model illustrated by the low pfu:LD50 ratio, a feature attributable to the expression of the Nipah G protein.

### Monkey neurovirulence test

Since the neurovirulence profile of PHV02 was not consistent across small animal species (mice and hamsters) and since PHV02 was more neurovirulent than rVSV-EBOV, further evaluation was undertaken in nonhuman primates (cynomolgus macaques). The selection of the control for the MNVT was based on several considerations. First, if a neurovirulence test is indicated, the test as performed should be able to distinguish reliably between acceptable and unacceptable preparations. Since no such reference strains exists, it was decided to use YF 17DD which has a well-defined pattern of histopathological changes, documented in many studies over many years using a standardized protocol for sectioning and scoring brain centers, against which PHV02 could be compared, and for which we could obtain a commercial source of well characterized virus. In contrast, there is only a single (non-GLP) study of rVSV-EBOV [35]. We were also unable to secure commercial Ervebo vaccine for use as a reference strain in the MNVT. Since PHV02 was more neurovirulent in infant mice than rVSV-EBOV, we were concerned that a comparison in monkeys could also show higher histopathological scores and that such a finding *in vacuō* could raise concerns for clinical use. On the other hand, if PHV02 were shown to be less neurovirulent that YF 17DD, which has a defined incidence of vaccine-associated neurotropic adverse events in humans, the neurovirulence profile would be deemed acceptable. Addition of another group of 10 monkeys in order to test the research grade rVSV-EBOV virus in a GLP study, while scientifically interesting, was considered unnecessary to achieve the goal of demonstrating nonclinical safety. These considerations for selection of the reference test article were subject to review by the US FDA, and the study rationale, study protocol and results were accepted.

The study design, dose and route of inoculation justifications are provided in **Table 1**. The MNVT was conducted according to current WHO requirements for YF vaccines [36].

**Table 1:** Design of the Monkey Neurovirulence Test.

Group	Treatment	Concentration of Dosed Formulation	Total Volume of Test Material to be Administered (mL)	Total Dose Administered	No. animals	
					Male	Female
1	PHV02	4 × 10^7^ pfu/mL	0.5^a^	2 × 10^7^ pfu^c^	5	6
2	YF 17DD	6.34 × 10^4^ IU/mL	0.25^b^	1.6 × 10^4^ IU^c^	6	5
3	10 mM Tris, 0.25% HSA^d^	0 pfu/mL	0.5^a^	0 pfu	1	2

pfu = plaque forming units; IU = International units

a. Total volume administered (0.25 mL in the right and left hemispheres by the intrathalamic route). The number of monkeys and the intrathalamic route for neurovirulence testing of live vaccines (other than polio) are specified by European Pharmacopoeia 5.0, 01/2005:2060, [2.6.18] Tests for neurovirulence of live virus vaccines, the WHO Expert Committee on Biological Standardization, 43rd Report, Technical Report Series 840, Geneva 1994; 45th Report; Annex 3 Requirements for measles, mumps and rubella vaccines and combined vaccine (live) (Requirements for Biological Substances No. 47)

b. Total volume administered in the left frontal lobe. The number of monkeys and the frontal lobe rote of inoculation are specified by the WHO Requirements for Yellow Fever vaccine, Technical Report Series, No. 872, 1998 and the Annex 5 updated requirements (2012).

c. The dose of PHV02 is the highest clinical human dose planned for administration by the intended (IM) route of inoculation. The dose of YF 17DD in 0.25 mL is determined by the concentration of full-strength reconstituted commercial lyophilized vaccine and exceeds the minimum dose requirement (1000 IU) as specified in the WHO Requirements, *op*. *cit*.

d. Vehicle control (buffer in which PHV02 drug product is suspended). HSA=recombinant human serum albumin.

All animals were seronegative antibodies to YF, Nipah and EBOV at baseline. All were confirmed to have received the intended test article by either demonstration of viremia by qRT-PCR [Group 1 (PHV02)] (**S5 Fig. PHV02 viremia determined by qRT-PCR in cynomolgus macaques by day after inoculation by the intrathalamic route with 2 x 10^7^ pfu of PHV02**), YF 17DD by plaque assay (**S1 Table. Yellow fever 17DD viremia)** or by development of antibodies (**S2 Table. Antibody responses, cynomolgus macaques Day 31 following IC inoculation of PHV02, YF 17DD or 0.9% saline**). Group 3 0.9% saline controls were negative for viremia and antibody. Viremia following IC inoculation in the MNVT is considered a measure of viscerotropism [30,36] since the inoculum is rapidly disseminated systemically and replicates in extraneural tissues. Viremia was detected in 11/11 (100%) of the animals inoculated with PHV02 on Day 3 and in 9 or 10/11 (81-90%) of the animals on Days 5 and 7, respectively, with peak geometric mean level of 4,866 copies/mL on Day 3 (**S5 Fig. PHV02 viremia determined by qRT-PCR in cynomolgus macaques by day after inoculation by the intrathalamic route with 2 x 10^7^ pfu of PHV02**). The highest individual value was 142,160 copies/mL on Day 7. Viremia was cleared in most animals by Day 15 and in all by Day 31. Viremia in the YF 17DD control group was measured by a less sensitive method (plaque assay) and was found at low titers (20- 140 pfu/mL) in 4/11 animals on Day 3 and 2/11 animals on Day 5 **(S1 Table Yellow fever 17DD viremia)**, well within WHO requirements [36] (**S1 Text “Viscerotropism” as Measured by Viremia Levels in the MNVT).**

Monkeys were closely observed between the day of inoculation and Day 31 when they were humanely euthanized and subsequently necropsied. Day 31 (30 days after inoculation) is the time for histopathological examination specified in the MNVT requirements [21,36]. There were no treatment-related changes in clinical signs or clinical laboratory measurements (**S2 Text Clinical findings, Cynomolgus Macaques, Monkey Neurovirulence Test).**

Histological examination of extraneural organs was limited to the liver, heart, kidneys, spleen, and adrenal glands. In addition, the eyes with optical nerves were examined. There were no microscopic lesions in any organ indicating viral multiplication or impaired function. Histological examination of the CNS was limited to the frontal cerebral cortex, diencephalon, mid-brain, brain stem nuclei, cerebellum, and cervical and lumbar cord enlargements. Neurovirulence was evaluated using the semiquantitative scoring system for inflammation and neuronal damage specified in the YF vaccine requirements [36]. Three separate scores were calculated: Discriminator area, Target areas, and Combined area (Discriminator plus target areas) [28,36]. Needle-tract microscopic changes at injection sites were observed in nearly all animals and were readily differentiated from virus-associated changes.

Monkeys inoculated with PHV02 showed no or minimal neuropathological changes; a few inflammatory lesions compatible with viral multiplication were observed in 3/11 animals (nos. 102, 105 and 151) limited to in the frontal cortices and amygdala and consisting of minimal, perivascular lymphoid cuffs and parenchymal aggregates of mononuclear cells (histopathology score ranged from 0.25 to 1.00 (**Table 2)**. The remaining 8/11 PHV02 monkeys had no microscopic lesions. Discriminator area (globus pallidus, putamen, thalamic nuclei), Target area (substantia nigra, cervical and lumbar cord enlargements), and combined group scores were 0.0.

**Table 2:** Monkey Neurovirulence Test: Individual Brain and Spinal Cord Scores^a^, Cynomolgus Macaques, evaluated 30 days after inoculation.

Animal ID	Frontal Cortex	Amyg-dala	Basal Ganglia			Thalamus		SN^b^	*Formatio Reticularis*			Spinal Cord Enlargement		
			N.Cau^b^	G.pall^b^	Puta^b^	N.ant./med^b^	N.lateral^b^		Pons	Medulla		Cervical		Lumbar
**Group 1: PHV02 Test Article; 2** × **10^7^ PFU**														
**101**	0.00	0.00	0.00	0.00	0.00	0.00	0.00	0.00	0.00	0.00			0.00	0.00
**102**	0.25	1.00	0.00	0.00	0.00	0.00	0.00	0.00	0.00	0.00			0.00	0.00
**103**	0.00	0.00	0.00	0.00	0.00	0.00	0.00	0.00	0.00	0.00			0.00	0.00
**104**	0.00	0.00	0.00	0.00	0.00	0.00	0.00	0.00	0.00	0.00			0.00	0.00
**105**	0.50	1.00	0.00	0.00	0.00	0.00	0.00	0.00	0.00	0.00			0.00	0.00
**151**	1.00	1.00	0.00	0.00	0.00	0.00	0.00	0.00	0.00	0.00			0.00	0.00
**152**	0.00	0.00	0.00	0.00	0.00	0.00	0.00	0.00	0.00	0.00			0.00	0.00
**153**	0.00	0.00	0.00	0.00	0.00	0.00	0.00	0.00	0.00	0.00			0.00	0.00
**154**	0.00	0.00	0.00	0.00	0.00	0.00	0.00	0.00	0.00	0.00			0.00	0.00
**155**	0.00	0.00	0.00	0.00	0.00	0.00	0.00	0.00	0.00	0.00			0.00	0.00
**156**	0.00	0.00	0.00	0.00	0.00	0.00	0.00	0.00	0.00	0.00			0.00	0.00
**Group 2: YF 17DD Reference Article; 1.6** × **10^4^ IU**														
**201**	1.50	2.00	2.00	1.75	2.00	0.00	2.00	2.00	1.50	1.00			0.58	1.08
**202**	1.75	2.00	2.00	1.50	1.50	0.00	1.50	1.00	1.00	1.50			0.50	0.67
**203**	1.50	1.50	1.50	1.25	1.50	1.00	2.00	1.50	1.00	1.50			0.75	0.42
**204**	1.50	1.50	2.00	1.25	1.25	1.00	1.00	2.00	1.00	1.00			0.92	0.83
**205**	1.50	1.50	1.50	1.25	0.75	0.50	1.00	2.00	0.50	0.00			0.08	0.25
**206**	1.25	1.50	1.50	0.75	1.25	0.50	0.75	2.00	0.00	1.00			1.17	0.25
**251**	1.00	2.00	2.00	2.00	2.00	0.00	2.00	2.00	1.00	1.00			1.67	1.58
**252**	0.75	2.00	2.00	1.50	1.50	1.50	1.00	2.00	1.00	1.00			0.67	0.25
**253**	1.75	2.00	2.00	0.75	2.00	1.00	1.50	1.50	1.00	1.00			0.83	0.75
**254**	1.00	2.00	2.00	1.50	1.75	0.00	0.00	1.50	0.00	0.00			0.08	0.00
**255**	0.75	0.00	0.00	0.50	0.75	0.00	0.00	1.00	0.00	0.00			0.17	0.00
**Group 3: Vehicle Control Article; 10 mM Tris, 0.25% HSA**														
**301**	0.00	0.00	0.00	0.00	0.00	0.00	0.00	0.00	0.00	0.00	0.00			0.00
**351**	0.00	0.00	0.00	0.00	0.00	0.00	0.00	0.00	0.00	0.00	0.00			0.00
**352R**	0.00	0.00	0.00	0.00	0.00	0.00	0.00	0.00	0.00	0.00	0.00			0.00

^a^ Histopathologic grades were assigned according to the following scale: minimal (1), mild (2), moderate (3), and marked (4), based on increasing extent and/or complexity of change, as described in Materials and Methods. Average scores for the right and left hemispheres are shown. The cervical and lumbar enlargements were each divided into 6 equal sections and the right (R) and left (L) hemisections for scoring.

^b^N.Cau = Nucleus Caudatus; G.pall = Globus Pallidus; Puta = Putamen; N.ant/med = Nucleus Anterior/Medial Thalami; SN = Substantia Nigra

In contrast, inflammatory lesions and neuronal loss were observed in all 11 monkeys inoculated with YF 17DD (**Table 2**). Although the virus was inoculated in the left frontal lobe, there was no difference observed in lesions in the left and right hemisections. Inflammatory lesions consisted of minimal to-moderate perivascular lymphoid cuffs and parenchymal aggregates of mononuclear cells (histopathology score 1 or 2). Neuronal satellitosis, degeneration, or necrosis were rare and mainly seen in lesions of moderate severity (histopathology score 2). The most commonly affected areas of the CNS were frontal cortices, basal ganglia (caudate nucleus, putamen, and globus pallidus), amygdala, thalamus (anterior/medial and lateral nuclei), substantia nigra, *formatio reticularis* (pons and medulla), and spinal cord enlargements. The individual average histopathology score for Discriminator areas varied from 0.31 to 1.50 (mean group score = 1.12) and for Target areas from 0.39 to 1.75, with a mean group score = 0.97) (**Table 3**). The combined Target and Discriminator mean group score was 1.06. The YF 17DD mean Discriminator, Target and combined scores were statistically higher than for PHV02 (p<0.0001, Wilcoxon rank sum). The vehicle control animals had no neuropathological lesions.

**Table 3:** Discriminator, Target, and Combined Scores, Cynomolgus Macaques, Neurovirulence Test, evaluated 30 days after inoculation.

Animal ID	Histopathology Scores		
	Discriminator Areas^a^	Target Areas^b^	Combined
**Group 1: PHV02 Test Article; 2 × 10^7^ pfu**			
**101**	0.00	0.00	0.00
**102**	0.00	0.00	0.00
**103**	0.00	0.00	0.00
**104**	0.00	0.00	0.00
**105**	0.00	0.00	0.00
**151**	0.00	0.00	0.00
**152**	0.00	0.00	0.00
**153**	0.00	0.00	0.00
**154**	0.00	0.00	0.00
**155**	0.00	0.00	0.00
**156**	0.00	0.00	0.00
**Group Mean (SD)**	**0.00 (0.00)**	**0.00 (0.00)**	**0.00 (0.00)**
**Group 2: YF 17DD Reference Article; 1.6 × 10^4^ IU**			
**201**	1.44	1.22	1.34
**202**	1.13	0.72	0.95
**203**	1.44	0.89	1.20
**204**	1.13	1.25	1.18
**205**	0.88	0.78	0.83
**206**	1.00	1.14	1.06
**251**	1.50	1.75	1.61
**252**	1.38	0.97	1.20
**253**	1.31	1.03	1.19
**254**	0.81	0.53	0.69
**255**	0.31	0.39	0.35
**Group Mean (SD)**	**1.12**^**c**^ **(0.36)**	**0.97^c^ (0.38)**	**1.06^c^ (0.34)**
**Group 3: Vehicle Control Article; 10 mM Tris, 0.25% HSA**			
**301**	0.00	0.00	0.00
**351**	0.00	0.00	0.00
**352R**	0.00	0.00	0.00
**Group Mean (SD)**	**0.00 (0.00)**	**0.00 (0.00)**	**0.00 (0.00)**

^a^ The discriminator areas include globus pallidus, putamen, and thalamic nuclei

^b^ The target areas include substantia nigra and both cervical and lumbar spinal cord enlargements.

^c^ p < 0.0001 vs. PHV02, Wilcoxon rank sum test

Representative histopathological changes in brain and spinal cord of monkeys inoculated with YF 17DD are provided in **S6 Fig Representative YF 17DD histology lesions** and **S7 Fig Representative YF 17DD histology lesions (cont.)** for YF 17DD and **S8 Fig Representative PHV02 histology lesions** for PHV02.

At termination of the study (Day 31), all Group 1 monkeys had developed IgG antibodies against NiV G with titers between 229 and 1005 EU/mL (geometric mean titer 563, **S2 Table Antibody responses, cynomolgus macaques Day 31 following IC inoculation of PHV02, YF 17DD or 0.9% saline**). All Group 2 monkeys had developed YF 90% plaque reduction neutralization (PRNT90) titers of 1:80–640 (geometric mean titer 300, **S2 Table Antibody responses, cynomolgus macaques Day 31 following IC inoculation of PHV02, YF 17DD or 0.9% saline**). Group 3 animals inoculated with 0.9% saline were seronegative (<1:20).

Results of the MNVT were filed to and enabled the Investigational New Drug (IND) application for clinical trials.

## Discussion

Using a reverse genetic system, a rVSV vector was constructed by removing the VSV glycoprotein (G) gene (VSVΔG) and pseudotyping the virus by inserting the desired foreign gene for inducing an immune response to the desired target, in this case NiV G protein [3]. A pseudotype expressing only the NiV G, responsible for cell attachment but not mediating fusion, did not produce replicating virus unless a fusion protein [F protein of NiV or the glycoprotein (GP) of Ebola] was co-expressed. Preliminary observations of clinical signs in hamsters suggested the vector expressing the NiV F protein may not be sufficiently attenuated. Therefore, rVSVΔG-EBOV GP/NiV G rather than rVSVΔG NiV F/G was selected for vaccine development, and the rVSV-NiV F/G virus was used only to measure specific NiV specific antibodies in a plaque reduction neutralization test. Studies comparing single cycle rVSV vectors expressing NiV G, NiV F, or both F and G proteins have shown equivalent protective activities [3,37]. Thus, inclusion of only NiV G is sufficient for effective immunization. Additionally, G protein subunit vaccine and G protein-specific monoclonal antibody to the closely related Hendra virus (HeV) are protective against NiV challenge in nonhuman primates [38]. Thus, the evidence strongly supports the protective activity of the G protein of NiV and inclusion in the PHV02 vector.

The Ebola GP is superfluous to the indication for prevention of Nipah virus disease but may blunt booster responses due to anti-vector immunity; fortunately, the evidence from the single-dose study of IM inoculation of PHV02 in hamsters reported here (as well previously reported studies in hamsters [3] and in African green monkeys [4]) suggests that PHV02, like Ervebo, will elicit protective immunity after a single dose and that pre-existing immunity to Ebola is not a factor limiting immunization in Asia where the rVSV-Nipah vaccine is required.

Safety is the principal concern for live vaccines, including recombinant vectors with virulence genes donated by the wild-type pathogen. The WHO guidance states that the need for a neurovirulence test should be based on evidence either that the natural infection is neurotropic [27], which is the case for both VSV [39] and NiV [40] (and possibly the EBOV [41]) components of the PHV02 vector. Fortunately, there is now an extensive nonclinical and clinical experience with the use of VSV vectors in which the principal virulence factor (the VSV G protein) has been deleted [26]. Most relevant, the live, attenuated rVSV-EBOV vaccine has been approved for human use and is the backbone of the PHV02 vaccine candidate [5,6]. rVSV-EBOV (Ervebo) has now been used in hundreds of thousands of people, with a good safety record [42]. However, the addition of the NiV G protein, which is involved in cell attachment, could potentially change tropism and virulence of the vector. In particular, the NiV G protein is a ligand for ephrin B2 and ephrin B3 receptors expressed on neurons and is believed to play a role in neurotropism and encephalitis in human NiV disease [43].

We initially studied the neurovirulence of the rVSV-Nipah candidate in small animal models. It may be acceptable to substitute a small animal for nonhuman primates if the model can be shown to be representative of the risk to humans [44]. In the case of PHV02, we prespecified in regulatory documents that an MNVT would be required in the event PHV02 was more neurovirulent in rodents than the authorized rVSV-EBOV vaccine. The virus stocks used in neurovirulence studies in mice, hamsters and nonhuman primates varied with respect to passage level (**S3 Table. Viruses used in neurovirulence and toxicity studies**) but were shown to be virtually identical by deep sequencing (see **Materials and Methods**). In addition to the neurovirulence studies, a biodistribution study in a ferret model showed no evidence for neuroinvasion; this study will be reported separately.

Since the rVSV-EBOV vaccine differs only in lacking NiV G, it was used as a control to dissect the role of the G protein in neuropathogenesis. The rVSV-Nipah vector IC LD50 in infant (8-day-old) mice was approximately 5 pfu, at least 20 x more neurovirulent than rVSV-EBOV (IC LD50 >100 pfu). Interestingly, we showed similar viral loads in mice that died after inoculation of 10 pfu PHV02 as in mice that survived after inoculation with rVSV-EBOV at up to 100 pfu (**S4 Fig. Viral load determined by qRT-PCR in brain and spleen tissues from Swiss-Webster mice**), possibly indicating that neurovirulence was mediated by mechanisms independent of viral replication level; however, this observation could also be explained by timing of the samples for viral load. The 8-day-old mouse has been used for studies of the comparative neurovirulence of other vaccines and was sensitive for detection of virulence changes caused by a single amino acid mutation [45]. Although infant mice are exquisitely sensitive to neurotropic viruses, mice develop resistance to direct IC inoculation with advancing age. By 6-8 weeks of age, mice fully survived IC inoculation of PHV02 vaccine and had minimal brain pathological changes. This was not the case, however, for another rodent (hamster) model in which both infant and adult animals were susceptible to encephalitis and death after IC inoculation of PHV02. Adult hamsters inoculated by the intended (IM) route with the vaccine, however, showed no clinical or histological signs of neuroinvasion. Other studies showed that adult hamsters inoculated IM developed low viremia levels of 4-6 log10 copies/mL, estimated to be 2-4 log10 pfu based on a ratio of copies:pfu for the qRT-PCR assay of 44.68 (**S1 Fig. Toxicity of PHV02 compared to wild-type VSV, Panel D**), levels that were probably incapable of crossing the blood-brain barrier.

rVSV-EBOV has been reported to lack neuropathologic changes in nonhuman primates inoculated by the intrathalamic route [35]. While no human cases of rVSV-EBOV vaccine-associated neurotropic adverse events have been reported, the clinical experience is still quite limited [42]. To further unravel the safety profile of the rVSV-Nipah vaccine candidate, we compared its neurovirulence to that of commercial YF 17DD vaccine (YF 17DD is one of 3 substrains used for manufacture of YF vaccines that include 17D-204, and 17D-213, and are considered to be biologically equivalent [46]). The test would compare the neurovirulence of PHV02 and YF 17DD vaccine, rather than rVSV-EBOV. The rationale for use of YF was based on two factors: (1) the MNVT for YF vaccines is a well-established method governed by international requirements with semi-quantitative endpoints capable of demonstrating relatively small differences in neurovirulence [28,32] and (2) YF 17D vaccine has a known reporting rate of neurotropic vaccine-related adverse events. In contrast, rVSV-EBOV, while a logical control because it is a similar vector lacking the NiV G transgene, has a much shorter track record in terms of human use. From a regulatory science perspective, it is not typical to use a completely different vaccine type as a reference control in toxicology studies. Notwithstanding, the rationale for use of a heterologous virus as a comparator and the test methods for the MNVT were prospectively accepted by the US FDA enabling regulatory submission for clinical testing of PHV02.

YF vaccine has been in use since 1937, with hundreds of millions of doses administered, and a large body of data on the occurrence of YF vaccine-associated neurotropic adverse events. YF vaccines retain a high degree of neurovirulence for infant and adult mice, an observation that was confirmed in the current studies. The degree of pathological changes in brains of nonhuman primates inoculated IC with different YF vaccine stocks manufactured before stabilization of passage level by a seed stock system correlated with the occurrence of post-vaccinal encephalitis in humans and nonhuman primates [47]. This ultimately led to use of a standardized MNVT for YF vaccine master and working viral seeds [28]. The test reveals a mild degree of neurovirulence by histopathological scoring and occasional clinical signs of encephalitis. The residual neurovirulence for mice and monkeys is associated with the occurrence of YF vaccine associated neurotropic disease (YEL-AND) in humans. The overall reporting rate in the US population estimated from the Vaccine Adverse Event Reporting System (VAERS) data is between 0.3 and 0.8 per 100,000 vaccinations [48–50]. The incidence of YEL-AND is higher at the extremes of age, with infants <6 months for whom the vaccine is contraindicated having the highest risk, followed by the elderly. We followed the procedures specified by WHO requirements for YF vaccine to compare PHV02 with a commercial lot of YF 17DD vaccine (**Materials and Methods**) [36]. This histopathological scoring system used in the YF MNVT is more comprehensive with respect to systematic scoring of lesions by brain center, compared to tests applied to other viral vaccines. Even though there are some specific aspects to the scoring system, as noted below, the brain centers scored are considered important in the assessment of any neurotropic virus. Discriminator area lesion scores are capable of differentiating a YF vaccine lot with higher neurovirulence compared to a reference lot, whereas Target areas are affected by all YF vaccine strains [28]. In our study, the MNVT showed a typical pattern of inflammatory and neuronal damage associated with YF 17 D vaccines in macaques [28], including the YF 17DD substrain [51], whereas PHV02 showed virtual absence of neurovirulence following intrathalamic administration. This suggests a very low potential for PHV02 to cause neurotropic adverse events in humans.

The absence of neurovirulence observed in the MNVT where the blood-brain barrier is by-passed by direct IC inoculation is reassuring. It is also important to assess the risk of replication in extraneural tissues and potential for neuroinvasion via the bloodstream. The ability of the vaccine virus to replicate in extraneural tissues (“viscerotropism”) is measured by virus in blood after IC inoculation since the inoculum is rapidly disseminated systemically. Viremia levels for both PHV02 and YF 17DD were shown to be within the established limits of the MNVT [36] and similar to published data for YF vaccines [46] (**S1 Text. “Viscerotropism” as Measured by Viremia Levels in the MNVT**) indicating limited viscerotropism and a low risk of neuroinvasion after parenteral inoculation of PHV02. In accordance with the viremia data indicating systemic infection, robust Nipah and YF specific immune responses followed IC inoculation in all animals in the respective treatment groups (**S2 Table Antibody responses, cynomolgus macaques Day 31 following IC inoculation of PHV02, YF 17DD or 0.9% saline).**

A previous analysis of neurovirulence of a recombinant viral vector indicated concordance of results between mouse and monkey models, suggesting that small animal model could potentially be used as a substitute for the MNVT 30]. Our study showed that this conclusion is not applicable, since discordant results between neurovirulence of PHV02 in rodents and monkeys were observed. The higher neurovirulence of PHV02 than rVSV-EBOV for infant mice and higher neurovirulence than YF 17DD for infant mice and infant and adult hamsters required further investigation in nonhuman primates. To that purpose we used a YF 17DD reference control with quantifiable neurotropism in nonhuman primates and a known reporting rate of neurotropic adverse events in humans. The results were definitive in showing a significantly lower level of neurovirulence for PHV02 than for YF 17DD vaccine, suggesting that the PHV02 vaccine will have a very low risk of neurotropic adverse events in humans. However, it should be emphasized that ultimately it is human clinical experience, not nonclinical studies, that defines vaccine safety, and that low-frequency adverse events may only come to light when several million doses of a new vaccine have been administered.

This study, using a heterologous reference virus, may establish a new approach for safety testing of other viral vaccines.

## Materials and methods

### Ethics statement

Protocols and study procedures were approved by the respective Institutional Animal Care and Use Committee (IACUC) and supervised by the veterinary services department where the study was performed. Animal care and housing met AAALAC International recommendations and current requirements [52]. The GLP nonhuman primate study and the non-GLP but well-documented hamster IM toxicity study required full documentation of housing, environmental conditions, food consumption and water.

### Animals

Initial studies of IC inoculations of mice and hamsters were performed by BioReliance Corporation, Rockville Maryland. Litters of outbred ICR mice (each with ten 2-day old or 8-day old suckling pups) and adult mice were sourced from ENVIGO, Frederick, Maryland. Litters of Golden hamsters (*Mesocricetus auratus*) each with 10 2-day old or 8-10 8-day old suckling pups and adult hamsters were sourced from ENVIGO, Indianapolis, Indiana. Confirmatory studies in 8 day-old and adult (6 to 8-week-old) mice were performed at the University of Texas Medical Branch (UTMB), Galveston Texas. Outbred Swiss Webster mice were obtained from Charles River Laboratories. The pilot study in adult hamsters was performed at UTMB. Methods are briefly described in the Section on **Toxicity of PHV02 by peripheral Inoculation and attenuation vs. wild-type VSV**. Clinical Score was assessed each time hamsters were observed. Scores were assigned to individual hamsters on a 0-5 scale for responsiveness, neurological signs of disease, general physical state and changes in weight. If a hamster reached a score of 5 in any one individual category or a sum score of greater than 13, the animal was humanely euthanized. The 28-Day Single-Dose Acute Toxicity, Local Tolerance, and Immunogenicity Study of PHV02 in Hamsters was conducted according to well-documented methods by AmplifyBio, West Jefferson, Ohio. Hamsters were obtained from Charles River Laboratories, Stone Ridge, NY and were approximately 8 weeks of age at initiation. The MNVT was also performed according to GLP by AmplifyBio under protocol “Single-Dose Neurovirulence Study of Recombinant Vesicular Stomatitis Nipah Vaccine and Yellow Fever 17DD Vaccine Active Control Following Intracerebral Administration to Cynomolgus Monkeys”. Animals (*Macaca fascicularis*) were sourced from Primate Products, Immokalee, Florida, quarantined for 27 days and acclimated to the laboratory environment. Monkeys were 2.41 to 2.71 years of age at group assignment.

### Viruses

Using a reverse genetic system, a rVSV vector was constructed by removing the VSV glycoprotein (G) gene (VSVΔG) and inserting the genes encoding EBOV GP and NiV G protein. This work was performed by the Feldmann Lab, Laboratory of Virology, Division of Intramural Research, NIAID, NIH as previously described [53]. Further details found in **S3 Text. Detailed Materials and Methods** (Vector Construction). The rescued virus, Passage (P) 1, was amplified in Vero E6 cells, and the P2 virus transferred to Ology Bioservices Inc, Alachua, Florida. The virus underwent 5 sequential rounds of plaque purification in certified Vero cells grown in serum/animal product free conditions and was then amplified in a fluid culture to P8. At each passage the virus underwent full genomic next generation sequencing (NGS). A single clone with fewest single nucleotide polymorphisms (SNP) (none in the NiV G gene) and good growth characteristics in Vero cells, was selected as the pre-master virus seed (pMSV, P8). The pMVS was used to produce a Master Virus Seed (MVS, P9), and, in sequence, Working Virus Seed (P10), and Vaccine lots (P11), thereby stabilizing the virus passage level for manufacturing. The seed viruses were stored (<-70°C) as clarified cell culture medium, but vaccine lots were purified a multistep process and formulated in 10mM Tris HCl pH 7.2 and 0.25% recombinant human serum albumin (HSA), and frozen as the final drug product. For the experiments described in this report, which were carried out at different times during development, different lots were used (**S3 Table. Viruses used in neurovirulence and toxicity studies).** Wild-type VSV Indiana strain L2-83 was obtained from the World Reference Center for Emerging Viruses and Arboviruses, University of Texas Medical Branch, Galveston Texas. VSV-EBOV was originally supplied by the Feldmann Laboratory (RML, NIAID) as clarified viral harvest (P2) from Vero cells and passaged once in Vero cells at Q2 (San Juan Capistrano, California) to produce a research viral stock.

#### Genetic stability

The sequences of the pre-Master Virus Seed and Master Virus Seed were 100% identical to the reference (P2 from original transfection), the Working Virus Seed was 99.99% identical, and the cGMP vaccine drug substance (P11) was 99.97% identical. There were no non-synchronous SNPs in the NiV G gene across passages. The WVS had 3 SNPs in VSV N and one in VSV L gene and the P11 vaccine had an additional non-synchronous SNP in the EBOV GP.

#### Antibody measurements

For plaque reduction neutralization tests (PRNT) performed in Vero cell monolayers against YF and NiV, virus stocks of YF (Asibi) and NiV (Malaysia) were used. IgG binding antibodies were measured by ELISA against purified recombinant NiV G protein. Detailed methods are provided in **Materials and Methods** and in **S3 Text. Detailed Materials and Methods** (Neutralizing antibody).

The lyophilized YF 17DD vaccine and diluent for reconstitution (water for injection) were supplied through the generosity of Dr. Akira Homma, FIOCRUZ, Fundação Oswaldo Cruz, Rio de Janeiro, Brasil. Both were commercial lots and were used within their expiry dates.

Saline (0.9%) for injection used as vehicle control in was sourced from commercial suppliers. Vaccine diluent (10 mM Tris HCl pH 7.2, 0.25% recombinant HSA in water for infection USP was manufactured for use in the MNVT by AmplifyBio, West Jefferson, OH.

Studies performed at Ology Bio confirmed that PHV02 virus could be diluted in 0.9% saline or 10 mM Tris HCl pH 7.2, 0.25% HSA and was stable under the conditions and timeframes used to perform the animal studies.

### Study and inoculation procedures

For the initial mouse and hamster study of neurovirulence at BioReliance Corporation, PHV02 dosing solutions were prepared by dilution in 0.9% saline for inoculation of 100,000, 1000, or 10 plaque forming units (pfu) in 20 μL. Lyophilized YF 17DD was reconstituted with manufacturer’s diluent (sterile water for injection) to yield a concentration of 3.18 x 10^4^ IU (pfu)/mL, for a dose of 640 pfu in 20 μL. Two litters of infant ICH mice or hamsters (2- and 8-days old) and 5 adult mice or hamsters were injected IC with 20 μL. Animals were observed for 21 consecutive days and clinical signs and deaths recorded.

Similar methods were used at UTMB to determine LD50 using a different range of doses of PHV02. Dosages were calculated such that adult animals would receive 20μL of 5.0 x 10^6^ pfu/mL (100,000 pfu) of each virus. Dosages for neonatal animals were prepared via a serial dilution in 0.9% saline for injection yielding 5.0 x 10^3^ pfu/mL, 5.0 x 10^2^ pfu/mL, 5.0 x 10^1^ pfu/mL, and 5.0 x 10^0^ pfu/mL, to be delivered in a 20μL dose (a final dose of 100, 10, 1, and 0.1 pfu, respectively). Adult female Swiss-Webster mice (n=10) were inoculated IC with 100,000 pfu of either PHV02 or rVSV-EBOV and 5 mice were inoculated with 0.9% saline. Five mice (2 mice from the 0.9% saline group) were weighed, then euthanized on day 5 post infection. Adult mice were perfused with 0.9% saline and brains and spleens placed in RNALater (Qiagen) and frozen for subsequent homogenization and determination of viral load by qPCR. Any mouse considered moribund was euthanized and recorded as dead for that day. Similar procedures were used for infant mice. For each dose group, 2 litters (16+ neonates) were inoculated with 20 mL of graded doses of PHV02, rVSV-EBOV and 1 litter was inoculated with negative control (0.9% saline). For the Day 5 necropsies of infant animals, perfusions were not performed. LD50 was calculated by the method of Reed and Muench [54].

The MNVT was performed in accordance with GLP regulations. For the MNVT, animals were assigned to dose groups using a randomization program stratified by gender. Personnel performing clinical assessments, euthanasia decisions, laboratory tests and pathological examinations were blinded to group assignment information.

Twenty-five (25) cynomolgus monkeys (12 male and 13 female) were screened negative for pre-existing neutralizing antibodies to YF, NiV, and EBOV and assigned to 1 of 3 dose groups (**Table 1**). Eleven animals received 2 x 10^7^ pfu of PHV02 (the highest dose intended for clinical trials) divided between two intrathalamic inoculations, of 0.25 mL, one per hemisphere. Eleven animals received 1.6 x 10^4^ IU of YF 17DD in 0.25 mL into the left frontal lobe.

For the YF 17DD dosing material the 10 x 0.5 mL dose lyophilized vaccine vial was reconstituted in a half-volume (2.5 mL) of manufacturer’s diluent (water for injection) so that a full human dose was suspended in the injection volume of 0.25 mL.

Negative control animals (n=3) received the 0.5 mL formulation buffer for PHV02 (10 mM Tris HCl pH 7.2, 0.25% HSA) divided between two intrathalamic inoculations, of 0.25 mL, one per hemisphere.

Anesthetized animals were placed prone on an inoculation table with a sighting device for alignment of the head along the horizontal and vertical axes. The skull was exposed by a small incision, and a small hole was drilled through the skull to enable inoculation of test article with a 22g x 3.5 inch needle. Animals were administered the PHV02 vaccine (Group 1) or formulation buffer (Group 3) via bilateral intrathalamic injections of 0.25 mL. The intrathalamic route is required for MNVT of new vaccines (other than polio and YF) [55]. A similar procedure was used for Group 2 inoculation (YF 17DD) except that the drill hole site for dose administration (0.25 mL) into the skull corresponded to the left frontal lobe as required by WHO [36].

Clinical observation methods are described in **S3 Text Detailed Materials and Methods** (Monkey Neurovirulence test, Clinical Observations).

On Study Day 31, animals were humanely terminated followed by exsanguination and immediately underwent sequential upper body perfusion, first with isotonic saline/5% acetic acid and then with neutral-buffered 10% formalin. After fixation, the brain and spinal cord were placed into a container with 10% neutral-buffered formalin. Sectioning and staining of the brain and spinal cord were performed according to procedures described below.

A 28-Day single-dose acute toxicity, local tolerance, and immunogenicity study of PHV02 was conducted by AmplifyBio in hamsters (*Mesocricetus auratus*), 8 weeks of age. The study used good documentation but was not performed under full GLP compliance. Groups of 40 animals (20 males, 20 females) were inoculated IM with PHV02 (3.04 x 10^7^ pfu/animal) or 0.9% saline). Half of the animals were sacrificed for pathological study at Day 15 (14 days after vaccination) and the remainder at Day 29.

### Anatomical and laboratory pathological examination

For the MNVT, all tissues specified by WHO for toxicological evaluation of vaccines [27] were collected at necropsy and preserved, but only selected tissues were processed for histopathology by hematoxylin and eosin (H&E) staining: eyes with optic nerve, adrenal glands (paired), heart, kidneys (paired), liver, lungs, and spleen. Brain and spinal cord were harvested, sectioned, stained with H&E and gallocyanin [32]. Numerical histopathology scores were given to each hemisection of the frontal lobe, corpus striatum and thalamus, mesencephalon, pons, medulla, cerebellum, and 6 equal sections and the right (R) and left (L) hemisections for scoring. An average score was calculated for each tissue section based using the following criteria: **0 (no changes); 1 (minimal**): 1-3 small, focal inflammatory infiltrates. A few neurons may be changed or lost; **2 (moderate)**: more extensive focal inflammatory infiltrates. Neuronal changes or loss affects not more than one-third of neurons; **3 (severe):** neuronal loss of 33-90% of neurons with moderate focal or diffuse inflammatory infiltrates; **4 (overwhelming):** more than 90% of neurons changed or lost, with variable but frequently severe, inflammatory lesions. Three separate histopathology scores were calculated for each monkey and then averaged: The Discriminator areas (globus pallidus, putamen, and thalamic nuclei) and Target areas (substantia nigra and both cervical and lumbar spinal cord enlargements) and combined Discriminator and Target area scores.

### Quantitative PCR

The quantitative Real-Time PCR (qRT-PCR) targets RNA of the N gene of the VSV vector. The assay is composed of three principal steps: (1) extraction of nucleic acids from the sample using the automated MagNA Pure 96 system, (2) parallel amplification and detection of VSV-N specific sequences using forward and reverse primers and probe for N sequence amplification at optimized concentrations and (3) quantification of the results of the real-time RT-PCR amplification. Amplification and detection are accomplished using TaqMan chemistry on the Applied Biosystems QuantStudio 6 Flex Real-Time PCR System (QS6). Further details of the qRT-PCR and controls used are given in **S3 Text. Detailed Materials and Methods** (Quantitative PCR). The assay is linear in the range of 500 to 1.0 x 10^7^ copies/mL and lower limit of quantification (LLOQ) was 400 copies/mL. The viral load determinations in mouse brain and spleen were performed without matrix interference studies.

### Plaque assay

YF 17DD viremia in cynomolgus macaques was determined by plaque assay of plasma samples in Vero (CCL-81) (American Type Culture Collection at passage 137) and grown in 12-well plates. Plaques were enumerated using immunochemical staining. The lowest number of plaques detected in multiple wells was 1 pfu. The LLOD for this assay was 20 pfu/mL. Details of the assay are provided in **S3 Text, Detailed Materials and Methods** (Plaque Assay for Viremic Samples).

### Neutralization tests

Neutralizing antibody titers were measured by plaque reduction methods in Vero cells. Sera were serially diluted twofold, mixed with virus, and incubated for 1 hour at 37C. After virus adsorption, monolayers were overlaid with methylcellulose to immobilize plaques, which were subsequently visualized by immunochemical staining. Details are provided in **S3 Text Detailed Materials and Methods** (Neutralizing Antibody).

### Nipah G protein IgG ELISA

The test is performed by Q^2^ Solutions using a fully qualified method. Microtiter plates are coated with purified recombinant NiV glycoprotein (The Native Antigen Company, Kidlington, UK). After blocking of the plate, samples and controls are incubated with the antigen coated wells. A reference standard (pooled sera from PHV02-vaccinated monkeys) is included along with High and Low Quality and Negative Control samples. Following washing, each well is then incubated with goat anti-human IgG conjugated to horseradish peroxidase. Chromogenic substrate is added to each well. The enzymatic reaction is stopped by addition of sulfuric acid. Interpolation of the standard curve is performed with 4 Parameter Logistic analysis. The reference monkey serum pool is used to calculate relative titers which are reported in ELISA Units (EU) per mL. The limits of Quantitation (LOQ) determined during assay qualification is 6.1-699.2 EU/mL, and samples <6.1 EU/mL are reported as <LLOQ. For a valid assay the High and Low Quality Controls and the Negative Quality control must be within a pre-determined acceptance range.

### Statistical methods

Survival times of mice and hamsters inoculated IC with different viruses or virus doses were compared by Kaplan-Meier estimates and log rank tests. Comparisons of mortality ratios were performed by Fisher’s exact test. Tests for a significant dose response based on mortality ratios used the Cochran-Armitage method. Testing for dose response on the survival distributions rather than on mortality incidence employed the Cox Proportional Hazards model. Adjustments for multiple comparisons were performed by Dunnett-Hsu test. Differences between treatment groups in viral loads were analyzed by a one-way ANOVA with a multiple comparison test. IN the MNVT, group mean scores for Groups 1 and 2 Discriminatory, Target, and combined Discriminator + Target histopathology scores were analyzed using the Wilcoxon Rank Sum (Mann Whitney U) test.

## Supporting information

S1 Fig Toxicity of PHV02 compared to wild-type VSV. (DOCX)

S2 Fig Neutralizing antibody titers against wild-type Nipah virus, toxicology study. (DOCX)

S3 Fig Body weights, 8-day-old Swiss-Webster mice by day after IC inoculation of graded doses of rVSV-Nipah (PHV02) or rVSV-EBOV. (DOCX)

S4 Fig Viral load determined by qRT-PCR in brain and spleen tissues from Swiss-Webster mice. (DOCX)

S5 Fig PHV02 viremia determined by qRT-PCR in cynomolgus macaques by day after inoculation by the intrathalamic route with 2 x 10^7^ pfu of PHV02. (DOCX)

S6 Fig Representative YF 17DD histology lesions. (DOCX)

S7 Fig Representative YF 17DD histology lesions (cont.). (DOCX)

S8 Fig Representative PHV02 histology lesion. (DOCX)

S1 Table Yellow fever 17DD viremia. (DOCX)

S2 Table Antibody responses, cynomolgus macaques Day 31 following IC inoculation of PHV02, YF 17DD or 0.9% saline. (DOCX)

S3 Table Viruses used in neurovirulence and toxicity studies. (DOCX)

S1 Text “Viscerotropism” as Measured by Viremia Levels in the MNVT. (DOCX)

S2 Text Clinical findings, Cynomolgus Macaques, Monkey Neurovirulence Test. (DOCX)

S3 Text Detailed Materials and Methods. (DOCX)
