# Supplementary material for: Recombinant vesicular stomatitis vaccine against Nipah virus has a favorable safety profile: Model for assessment of live vaccines with neurotropic potential
Source: PLoS Pathog. 2022 Jun 27;18(6):e1010658. doi: 10.1371/journal.ppat.1010658 (PMC9269911; doi:10.1371/journal.ppat.1010658)
Supplement: S1 Fig — (DOCX) [file ppat.1010658.s001.docx]

**1 Fig**

**A**


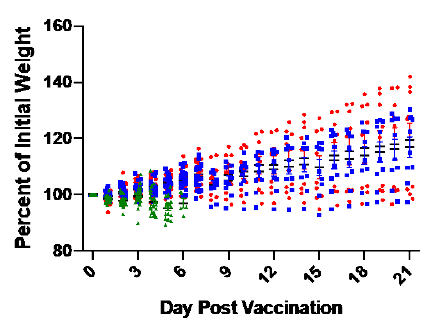

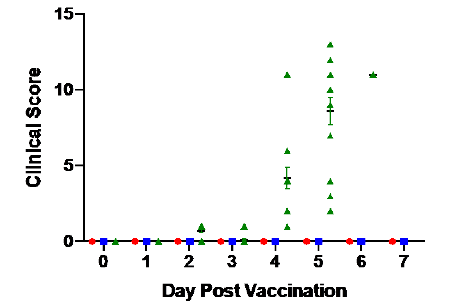


**C**

**B**


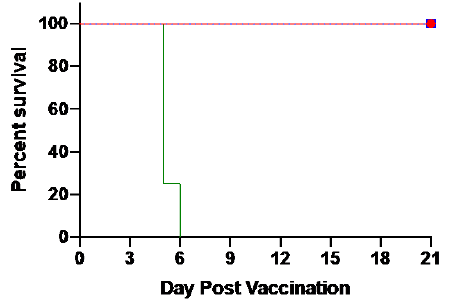

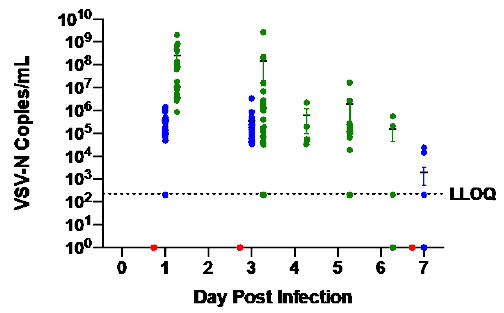


**D**

**S1 Fig. Toxicity of PHV02 compared to wild-type VSV**, non-GLP pilot study. Groups of ten 8-weekold hamsters were inoculated either rVSV-Nipah (PHV02 1.06 x 10^7^ /animal), wild-type VSV Indiana strain L2-83 (1.9 x 10^7^ pfu/animal) or 0.9% saline.

1. % change in body weight over the 21 day course of the study. Most animals inoculated with PHV02 (blue) or saline (red) gained weight, whereas hamsters inoculated IM with wild-type VSV (green) failed to gain or lost weight and were euthanized because of illness by day 6. Bars denote means ± SE. The variability in %weight change from day 7-21 is explained by gender differences, with females gaining weight more rapidly than males (data not shown)
2. Clinical scores on a severity scale of 0-5 for each of the following criteria on days 0-7: responsiveness/lethargy, neurological signs (twitching, altered movement, paresis/paralysis), hunching/ruffling, and % weight loss. Hamsters inoculated with PHV02 (blue) or saline (red) showed no signs of illness. Hamsters inoculated with wild-type VSV (green) had high clinical scores and died or were euthanized by day 6.
3. Survival distribution hamsters inoculated IM with PHV02 (blue); saline (red); wild-type VSV (green).
4. Viremia determined by RT-PCR. On day 1 peak levels were significantly higher in the wild-type VSV group (green) than in the PHV02 treatment (blue) group (p=0.027, *t* test 2-tailed). Viremia levels for VSV animals were higher on day 3 post inoculation, but differences did not reach statistical significance. Since animals in the VSV group showed illness, unplanned viremia data were obtained on days 4-6. Mean is shown as a horizontal line and error bars denote SE.
