# Supplementary material for: Recombinant vesicular stomatitis vaccine against Nipah virus has a favorable safety profile: Model for assessment of live vaccines with neurotropic potential
Source: PLoS Pathog. 2022 Jun 27;18(6):e1010658. doi: 10.1371/journal.ppat.1010658 (PMC9269911; doi:10.1371/journal.ppat.1010658)
Supplement: S2 Fig — (DOCX) [file ppat.1010658.s002.docx]

**Day 15 Day 29**


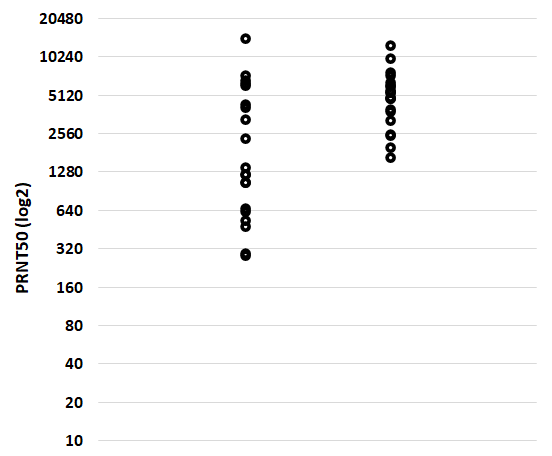


**S2 Fig.**. **Neutralizing antibody titers against wild-type Nipah virus, toxicology study.** Groups of ten (10) 8-weekold male and 10 female hamsters inoculated IM with 3.04 x 10^7^ pfu of rVSV-Nipah (PHV02) were tested on day 15 and groups of 10 male and 10 female were tested on day 29 by plaque reduction neutralization test (PRNT; PRNT50 = 50% neutralization titer) Individual animal titers are displayed. On Day 15 the geometric mean titer (horizontal bar) was 1766 (95% CI 1023, 3050) and significantly increased by Day 29 [GMT 4721 (95% CI 3700, 6023), p=0.0402 (*t* test with Welsh’s correction]. The day 0 titers were not determined, but all 20 male and female hamsters treated with 0.9% saline and tested on Day 15 and 10 male and female hamsters tested on day 29 were negative (PRNT50 <10).
