# Supplementary material for: Recombinant vesicular stomatitis vaccine against Nipah virus has a favorable safety profile: Model for assessment of live vaccines with neurotropic potential
Source: PLoS Pathog. 2022 Jun 27;18(6):e1010658. doi: 10.1371/journal.ppat.1010658 (PMC9269911; doi:10.1371/journal.ppat.1010658)
Supplement: S3 Fig — (DOCX) [file ppat.1010658.s003.docx]

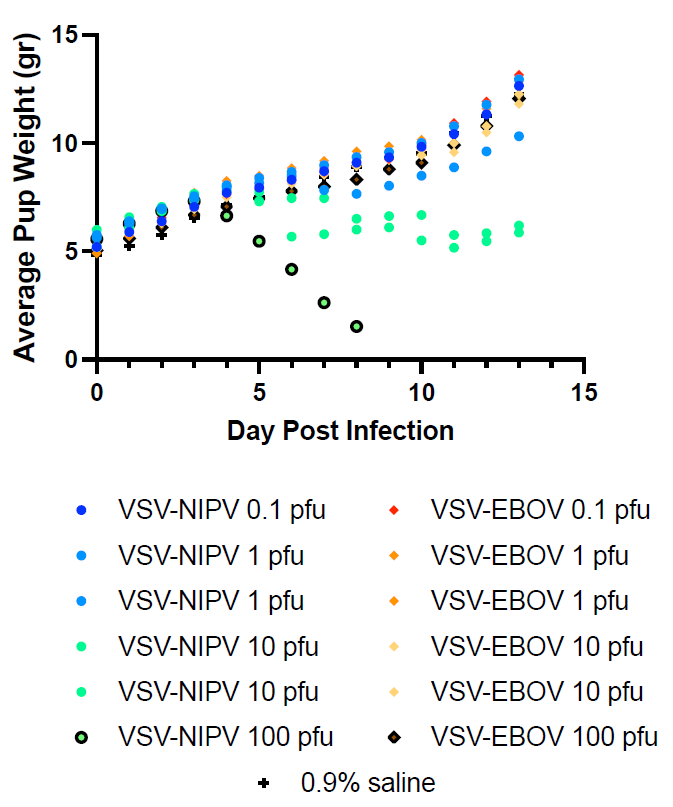


**S3** **Fig. Body weights, 8-day-old Swiss-Webster mice by day after IC inoculation of graded doses of rVSV-Nipah (PHV02) or rVSV-EBOV**. One litter of mice (10 pups each) was inoculated with 0.1 or 100 pfu, and two litters (10 pups each) were inoculated with 1 or 10 pfu. Mean weight of pups within a litter are shown by treatment and dose. PHV02 (designated VSV-NIPV) at 10 and 100 pfu resulted in death or failure to thrive and reduced weight gain in a dose-dependent fashion, whereas rVSV-EBOV was avirulent and body weights were similar to the 0.9% saline control. PHV02 mean pup weights were statistically lower (p<0.05, *t* test) than the rVSV-EBOV treated mice from day 6 to day 13. There was no statistical difference between the rVSV-EBOV and 0.9% saline controls.
