# Supplementary material for: Recombinant vesicular stomatitis vaccine against Nipah virus has a favorable safety profile: Model for assessment of live vaccines with neurotropic potential
Source: PLoS Pathog. 2022 Jun 27;18(6):e1010658. doi: 10.1371/journal.ppat.1010658 (PMC9269911; doi:10.1371/journal.ppat.1010658)
Supplement: S4 Fig — (DOCX) [file ppat.1010658.s004.docx]

## **
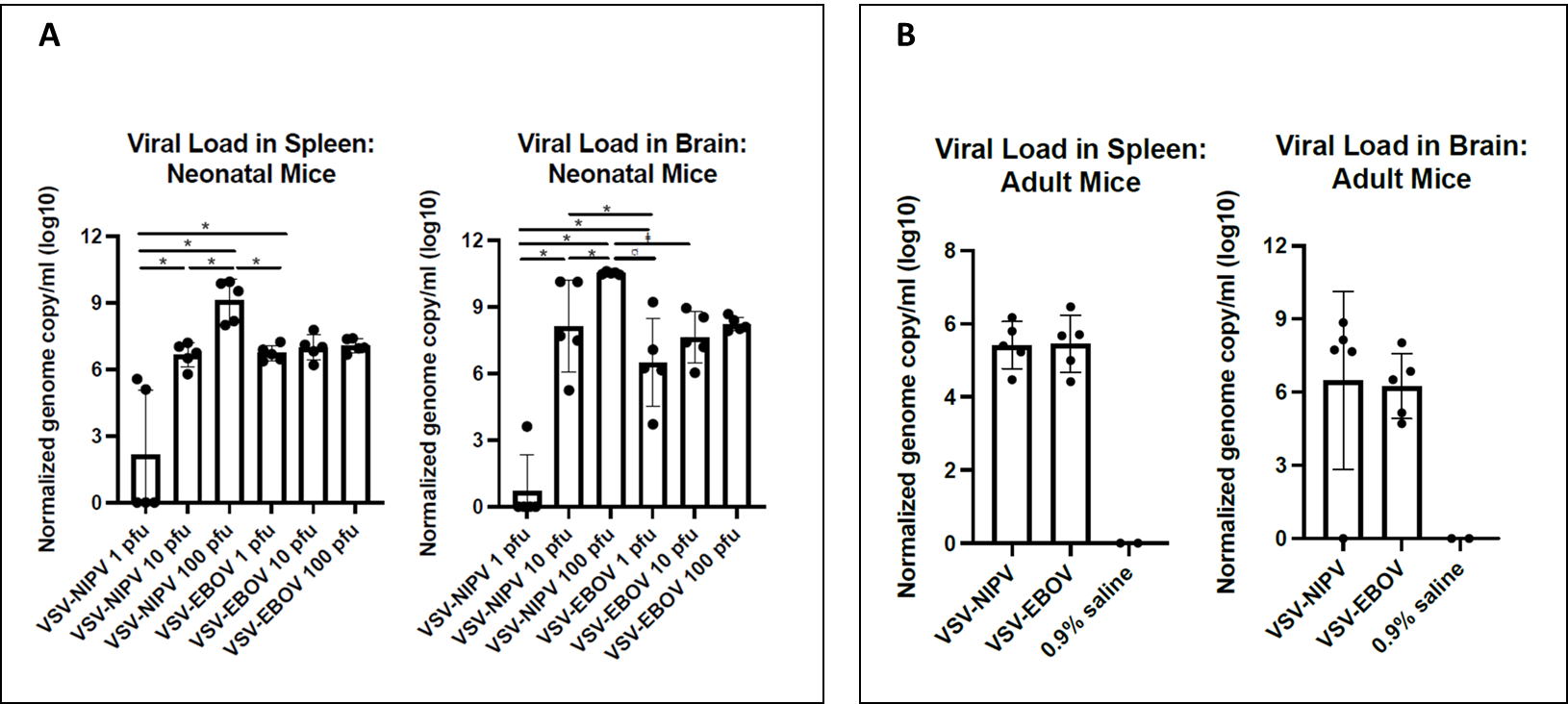
**

## **S4 Fig.** **Viral load determined by qRT-PCR in brain and spleen tissues from Swiss-Webster mice**

1. 8-day-old mice inoculated with graded doses of PHV02 or rVSV-EBOV; 5 mice per group were tested on Day 5 after IC inoculation with 1, 10 or 100 pfu of rVSV-Nipah or rVSV-EBOV. The histogram shows mean values and error bars denote standard deviation. Any value with a Ct > 38.68 was listed as negative and received a titer of 0 copies/mL in the graphs and for statistical analysis. Selected statistical differences shown: * p <0.001, 1-way ANOVA with multiple comparison; ¤ p=0019; ⱡ p=0.0387.
2. Adult mice inoculated with 100,000 pfu of PHV02 or rVSV-EBOV and tested on Day 5. Adult mice were perfused with 0.9% saline to remove blood before harvesting tissues for qRT-PCR. Parameters shown are as for infant mice. The differences between rVSV-Nipah and rVSV-EBOV viral loads were not statistically significant
