# Supplementary material for: Recombinant vesicular stomatitis vaccine against Nipah virus has a favorable safety profile: Model for assessment of live vaccines with neurotropic potential
Source: PLoS Pathog. 2022 Jun 27;18(6):e1010658. doi: 10.1371/journal.ppat.1010658 (PMC9269911; doi:10.1371/journal.ppat.1010658)
Supplement: S5 Fig — (DOCX) [file ppat.1010658.s005.docx]

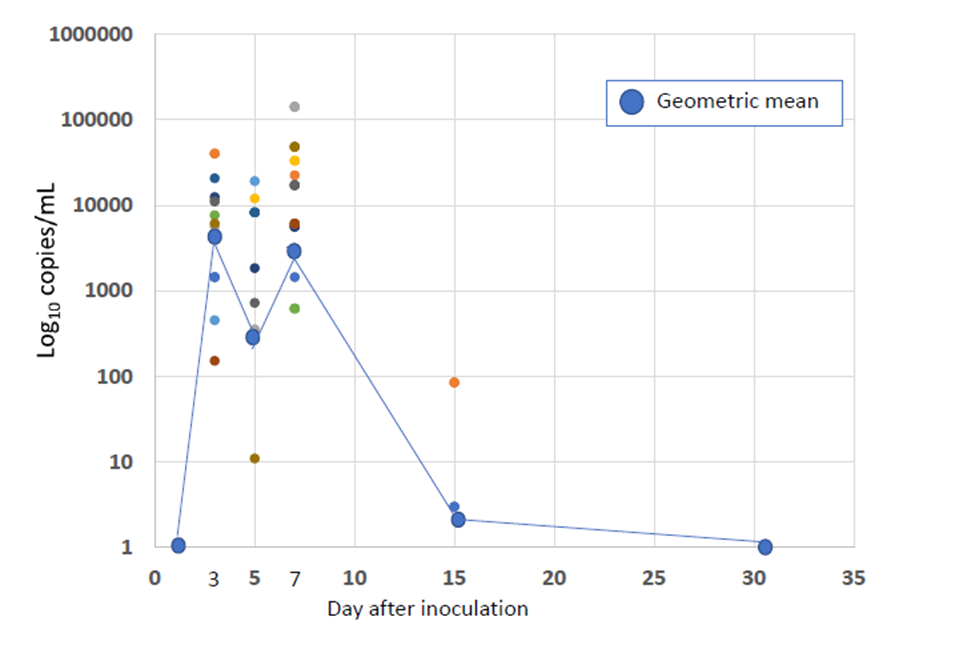


**S5 Fig.** **PHV02 viremia determined by qRT-PCR in cynomolgus macaques by day after inoculation by the intrathalamic route with 2 x 10^7^ pfu of PHV02**
