# Supplementary material for: Recombinant vesicular stomatitis vaccine against Nipah virus has a favorable safety profile: Model for assessment of live vaccines with neurotropic potential
Source: PLoS Pathog. 2022 Jun 27;18(6):e1010658. doi: 10.1371/journal.ppat.1010658 (PMC9269911; doi:10.1371/journal.ppat.1010658)
Supplement: S6 Fig — (DOCX) [file ppat.1010658.s006.docx]

**S6 Fig.** **Representative YF 17DD histology lesions**. **Panel A****.** Frontal cerebral cortex with inflammatory lesions (circle, black arrows) adjacent and distant to the inoculation site (blue arrow). **Panel B** higher magnification of Panel A showing large perivascular cuff of mononuclear cells and gemistocytes in the neuropils (small arrows); Score 2. Gemistocytes are reactive glial cells and indicate that inflammation has been present for some time. **Panel C.** Putamen and Globus Pallidus with numerous perivascular cuffs (arrow). **Panel D** higher magnification of Panel C depicting prominent mononuclear cell infiltrate around blood vessels spreading into the neuropil (arrow); Score 2. **Panel E.** Thalamic nuclei with aggregates of perivascular inflammation (circle, arrow) **Panel F** higher magnification of Panel E showing perivascular cuff with densely packed layers of mononuclear cells and an increase in the number of glial cells over the affected area; few gemistocytes (small arrows); Score 2. Hematoxylin and eosin staining was used. Original magnification X20 (Panels A, C, and E) and X400 (Panels B, D, and F).
