# Supplementary material for: Recombinant vesicular stomatitis vaccine against Nipah virus has a favorable safety profile: Model for assessment of live vaccines with neurotropic potential
Source: PLoS Pathog. 2022 Jun 27;18(6):e1010658. doi: 10.1371/journal.ppat.1010658 (PMC9269911; doi:10.1371/journal.ppat.1010658)
Supplement: S7 Fig — (DOCX) [file ppat.1010658.s007.docx]

**Fig S7.** **Representative YF 17DD histology lesions** **(continued).** **Panel A.** Substantia nigra illustrating several perivascular cuffs (arrows). **Panel B** higher magnification of Panel A showing perivascular cuff with densely packed layers of mononuclear inflammatory cells and few gemistocytes; Score 2. **Panel C.** Spinal cord with bilateral inflammatory aggregates in the ventral horns (arrows). **Panel D** higher magnification of Panel C viewing large dense mononuclear inflammatory cells around blood vessels spilling into the neuropil (arrow); Score 2. Hematoxylin and eosin staining was used. Original magnification X20 (Panels A and C) and X400 (Panels B and D).
