# Supplementary material for: Recombinant vesicular stomatitis vaccine against Nipah virus has a favorable safety profile: Model for assessment of live vaccines with neurotropic potential
Source: PLoS Pathog. 2022 Jun 27;18(6):e1010658. doi: 10.1371/journal.ppat.1010658 (PMC9269911; doi:10.1371/journal.ppat.1010658)
Supplement: S8 Fig — (DOCX) [file ppat.1010658.s008.docx]

**Fig S8**. **Representative PHV02 histology lesions.** **Panel A.** Frontal cerebral cortex with a single inflammatory lesion (arrow). **Panel B** higher magnification of Panel A showing a thin perivascular cuff of mononuclear cells; Score 1; three animals out of 11 inoculated with PHV02 had similar lesions in the frontal cerebral cortex. **Panel C.** Putamen and Globus Pallidus section with no lesions; Score 0. **Panel D.** Thalamic nuclei section with no lesions; Score 0. Substantia nigra section (arrows) with no lesions; Score 0**. Panel E**. Spinal cord section with no lesions; Score 0. Hematoxylin and eosin staining was used. Original magnification X20 (Panels A, B, C, D, E, and F).
