# Supplementary material for: Recombinant vesicular stomatitis vaccine against Nipah virus has a favorable safety profile: Model for assessment of live vaccines with neurotropic potential
Source: PLoS Pathog. 2022 Jun 27;18(6):e1010658. doi: 10.1371/journal.ppat.1010658 (PMC9269911; doi:10.1371/journal.ppat.1010658)
Supplement: S1 Table — (DOCX) [file ppat.1010658.s009.docx]

**S1 Table. Yellow fever 17DD viremia** determined by Vero cell plaque assay in cynomolgus macaques by day following IC inoculation of YF 17DD virus 1.6 x 10^4^ IU or 0.9% saline. The lower limit of detection (LOD) of the assay is 2.00E+01 pfu/mL.

| **Animal ID** | **Viremia (pfu/mL) by plaque assay in Vero cells and Day after inoculation** | | | | |
| --- | --- | --- | --- | --- | --- |
|  | **Day 1** | **Day 3** | **Day 5** | **Day 7** | **Day 15** |
| **Group 2: YF 17DD Reference Article; 1.6 × 10^4^ IU** | | | | | |
| **201** | <LOD | <LOD | **1.00E+02** | <LOD | <LOD |
| **202** | <LOD | <LOD | <LOD | <LOD | <LOD |
| **203** | <LOD | **2.00E+01** | **2.00E+01** | <LOD | <LOD |
| **204** | <LOD | <LOD | <LOD | <LOD | <LOD |
| **205** | <LOD | <LOD | <LOD | <LOD | <LOD |
| **206** | <LOD | **6.00E+01** | <LOD | <LOD | <LOD |
| **251** | <LOD | <LOD | <LOD | <LOD | <LOD |
| **252** | <LOD | **1.40E+02** | <LOD | <LOD | <LOD |
| **253** | <LOD | <LOD | <LOD | <LOD | <LOD |
| **254** | <LOD | **2.80E+01** | <LOD | <LOD | <LOD |
| **255** | <LOD | <LOD | <LOD | <LOD | <LOD |
| **Group 3: Vehicle Control Article; 10 mM Tris, 0.25% HSA** | | | | | |
| **301** | <20 | <LOD | <LOD | <LOD | <LOD |
| **351** | <20 | <LOD | <LOD | <LOD | <LOD |
| **352R** | <20 | <LOD | <LOD | <LOD | <LOD |
