# Supplementary material for: Recombinant vesicular stomatitis vaccine against Nipah virus has a favorable safety profile: Model for assessment of live vaccines with neurotropic potential
Source: PLoS Pathog. 2022 Jun 27;18(6):e1010658. doi: 10.1371/journal.ppat.1010658 (PMC9269911; doi:10.1371/journal.ppat.1010658)
Supplement: S2 Table — (DOCX) [file ppat.1010658.s010.docx]

**S2 Table.** **Antibody responses, cynomolgus macaques Day 31 following IC inoculation of PHV02,** **YF 17DD or 0.9% saline**. On Day 1, before inoculation, all animals in Groups 1-3 were seronegative for Nipah IgG antibodies (<LLOQ EU/mL) and for YF neutralizing antibodies (<20).

| **Animal ID** | **Day 31 Yellow fever PRNT_90_ (reciprocal titer)** | **Day 31 Nipah IgG ELISA (EU/mL)** |
| --- | --- | --- |
| **Group 1: PHV02 Test Article; 2 × 10^7^ pfu** | | |
| **101** | NT ^a^ | 609.6 |
| **102** | NT | 385.9 |
| **103** | NT | 802.6 |
| **104** | NT | 229.0 |
| **105** | NT | 345.8 |
| **151** | NT | 646.0 |
| **152** | NT | 974.8 |
| **153** | NT | 1004.8 |
| **154** | NT | 539.6 |
| **155** | NT | 483.6 |
| **156** | NT | 735.6 |
| **Geometric mean** |  | **563.4** |
| **Group 2: YF 17DD Reference Article; 1.6 × 10^4^ IU** | | |
| **201** | 160 | NT |
| **202** | 160 | NT |
| **203** | 640 | NT |
| **204** | 320 | NT |
| **205** | 160 | NT |
| **206** | 640 | NT |
| **251** | 640 | NT |
| **252** | 320 | NT |
| **253** | 320 | NT |
| **254** | 80 | NT |
| **255** | 640 | NT |
| **Geometric mean** | **300** |  |
| **Group 3: Vehicle Control Article; 10 mM Tris, 0.25% HSA** | | |
| **301** | <20 | <LLOQ |
| **351** | <20 | <LLOQ |
| **352R** | <20 | <LLOQ |
| **Geometric mean** | **<20** | <LLOQ |

*NT= not tested
