# Supplementary material for: Recombinant vesicular stomatitis vaccine against Nipah virus has a favorable safety profile: Model for assessment of live vaccines with neurotropic potential
Source: PLoS Pathog. 2022 Jun 27;18(6):e1010658. doi: 10.1371/journal.ppat.1010658 (PMC9269911; doi:10.1371/journal.ppat.1010658)
Supplement: S3 Table — (DOCX) [file ppat.1010658.s011.docx]

**S3 Table.** **Viruses used in neurovirulence and toxicity studies**

| **Experiment** | **Virus** | **Plaque purification** | **Manufacturer, Lot** | **Passage level* (Vero cells), titer** |
| --- | --- | --- | --- | --- |
| Pilot neurovirulence, infant/adult mice and hamsters | rVSV-Nipah. Clarified Vero cell culture harvest | No | Ology Bio Inc. | P3, 4.2 x 10^7^ pfu/mL |
| Confirmatory neurovirulence, infant/adult mice, adult hamsters | rVSV-Nipah Master Virus Seed (cGMP)  rVSV-EBOV. Clarified Vero cell culture harvest | Yes  No | Ology Bio Inc. Lot 28893  Q^2^ Solutions, Lot 05720 | P9, 5 x 10^7^ pfu/mL  P4, 3.1 x 10^7^ pfu/mL |
| Monkey neurovirulence | rVSV-Nipah Drug Product. cGMP vaccine lot | Yes | Ology Bio Inc. Lot 46982 | P11, 5 x 10^8^ pfu/mL |
| Comparative toxicity, PHV02 vs. wild-type VSV | rVSV-Nipah. Clarified Vero cell culture harvest  Wild-type VSV Indiana L2-83 | No  No | Ology Bio Inc.  UTMB  Lot TVP381 | P3, 4.2 x 10^7^ pfu/mL  4.3 x 10^8^ pfu/mL |
| Toxicity, hamster, intramuscular route | rVSV-Nipah non-GMP development lot | Yes | Ology Bio Inc. Lot PTR 3.23 | P11, 1.52 x 10^8^ pfu/mL |

*P0 is virus rescued from original transfection.
