# Supplementary material for: Recombinant vesicular stomatitis vaccine against Nipah virus has a favorable safety profile: Model for assessment of live vaccines with neurotropic potential
Source: PLoS Pathog. 2022 Jun 27;18(6):e1010658. doi: 10.1371/journal.ppat.1010658 (PMC9269911; doi:10.1371/journal.ppat.1010658)
Supplement: S1 Text — (DOCX) [file ppat.1010658.s012.docx]

**S1 Text “Viscerotropism” as Measured by Viremia Levels in the MNVT**

The WHO MNVT protocol specifies that viscerotropism of 17D (and 17DD) vaccine virus represented by circulating virus should not exceed 5E+02 IU (pfu) in 0.03mL of serum (i.e. 1.65E+04 IU/mL) and in no more than 1/10 monkeys should it exceed 1E+02 IU/0.03 mL (3.3E+03 IU/mL) [35]. In our study, viral RNA was detectable in blood of all animals inoculated with PHV02 with a peak geometric mean titer on Day 3 of 4,866 copies/mL (**S5 Fig.)**. The ratio of copies:pfu for the qRT-PCR assay was determined to be 44.68 indicating that geometric mean infectious viremia was less than ~1E+02 pfu/mL. The highest level in any one animal was 142,160 copies/mL, estimated to be 3.2E+03 pfu/mL). Viremia determined by plaque assay in animals inoculated with YF 17DD was only detected in 5/11 animals, with the highest titer being 1.4E+02 pfu/mL (**S1 Table**). Thus, viremia levels for both PHV02 and YF 17DD are within the established limits of the MNVT and similar to published data for YF vaccines [46], indicating limited viscerotropism and a low risk of neuroinvasion after parenteral inoculation. In accordance with the viremia data indicating systemic infection, a robust Nipah and YF specific immune response followed IC inoculation in all animals in the respective treatment groups (**S2 Table**.)
