# Supplementary material for: Recombinant vesicular stomatitis vaccine against Nipah virus has a favorable safety profile: Model for assessment of live vaccines with neurotropic potential
Source: PLoS Pathog. 2022 Jun 27;18(6):e1010658. doi: 10.1371/journal.ppat.1010658 (PMC9269911; doi:10.1371/journal.ppat.1010658)
Supplement: S2 Text — (DOCX) [file ppat.1010658.s013.docx]

**S2 Text. Clinical findings, Cynomolgus Macaques, Monkey Neurovirulence Test**

Monkeys were closely observed between the day of inoculation and Day 31. Clinical abnormalities were noted in 3 animals, 1 in each treatment group (PHV02, YF 17DD, 0.9% saline). One animal in the PHV02 group was noted to have intermittent bilateral mydriasis, not associated with other clinical findings and no pathological abnormalities in eye or brain on subsequent necropsy. One animal in the YF 17 DD group developed transient emesis, right forelimb weakness, whole body tremors, and excessive scratching of head on Days 1-3 after inoculation. One control animal developed emesis on Days 1 and 2. The clinical findings in the YF 17DD and control animals occurred in close temporal relationship to inoculation and were ascribed to inoculation trauma. There were no treatment related changes in body weight or food consumption. There were no treatment emergent hematologic abnormalities. Increases in serum transaminases (AST, ALT) were seen in all three treatment groups including the 0.9% saline controls and were ascribed to trauma and stress of the study procedures.
