# Supplementary material for: Recombinant vesicular stomatitis vaccine against Nipah virus has a favorable safety profile: Model for assessment of live vaccines with neurotropic potential
Source: PLoS Pathog. 2022 Jun 27;18(6):e1010658. doi: 10.1371/journal.ppat.1010658 (PMC9269911; doi:10.1371/journal.ppat.1010658)
Supplement: S3 Text — (DOCX) [file ppat.1010658.s014.docx]

**S3 Text DETAILED MATERIALS AND METHODS**

**Vector construction.** The expression vector for construction of the recombinant virus from cDNA contains the ColE1 origin of replication and amp resistance gene for selection. Into this vector, the genome of VSV (Indiana) was cloned, minus its glycoprotein (G) gene. The VSV genome expression was under the control of a T7 polymerase promoter. The coding sequence for Ebola EBOV (Mayinga, ZEBOV76GP) was inserted the VSV∆G backbone. Nipah virus Bangladesh consensus G was cloned into XhoI-NheI insertion site of the vector. ZEBOV76GP was removed by restriction digest and replaced with ZEBOV95GP (Kikwit) (representative of the Ervebo® vector). The Nipah NiV G gene was chemically synthesized by Invitrogen GeneArt Synthesis. A consensus Nipah NiV G sequence was constructed based on all full length NiV, Bangladesh genotype G protein sequences available from GenBank at the time. The recombinant plasmid was transfected in BHK-T7-9 cells expressing T7 polymerase.

**Monkey Neurovirulence test, Clinical Observations.**

Observations for clinical illness and mortality were performed twice daily during the quarantine and study periods, except on the day of necropsy when animals were only observed once. Cage-side clinical observations were performed on all animals assigned to study at least twice daily (in the morning and afternoon), from Day -7 to Day 31.

Signs of encephalitis, such as paresis, incoordination, lethargy, tremors, or spasticity, were assigned numerical values for severity and each animal was given a daily numerical score, based on the following grading method: 0 – No clinical signs of encephalitis; 1 – Rough coat, not eating; 2 – High pitched voice, inactive, slow moving; 3 – Shaky movements, tremors, incoordination, limb weakness; 4 – Inability to stand, limb paralysis, moribund, or dead. As the animals were examined twice daily, the higher score for the day was reported. The mean daily clinical score for each animal was the average of the animal’s daily scores, and the group mean daily clinical score was the average of the individual animal clinical scores for each day. Clinical score results are not presented in tabular format as there were only 3 clinical observations (one per treatment group and determined unrelated to treatment).

Individual body weights were recorded for all animals at least once prior to Day 1 for group assignment and for all animals assigned to study prior to dosing on Day 1 and on Days 7, 15, and 31. Daily food consumption was visually assessed. Blood specimens were collected for clinical pathology, viremia, and immunogenicity evaluations from all animals under ketamine anesthesia via the femoral vessel. On Day 30, animals were fasted overnight. Clinical pathology studies supporting both the adult hamster and nonhuman primate studies at AmplifyBio used validated hematological and clinical chemistry methods.

**Quantitative PCR.**

Quantification of RT-PCR results is achieved using an external standard curve of VSV-NiV-G calibrators. To verify RNA extraction from the specimen and successful RT-PCR amplification, a MS2 phage internal control (IC) is spiked into each sample prior to nucleic acid extraction and is amplified in parallel with the VSV-N target. Each MagNA Pure extraction run includes a High Positive Control (HPC), a Low Positive Control (LPC) and a Negative Control (NC). One pfu of PHV02 was estimated to be equivalent to 44.68 copies. The qPCR performed for the detection of viral loads in mouse brain and spleen and viremia in the MNVT was performed by Q2 Solutions, San Juan Capistrano, CA according to a method performed in monkey plasma substrate that had been qualified for linearity, intermediate precision, repeatability and sensitivity. The assay repeatability and intermediate precision was in acceptable ranges (CV% 15-56%).

**Plaque assay for viremic samples (YF 17DD).**

Vero cells were maintained in a 37°C incubator with 5% CO_2_ in culture media consisting of Dulbecco’s Modification of Eagle Medium (DMEM, Gibco, Waltham MA) supplemented to 10% vol/vol heat inactivated fetal bovine serum (FBS, Atlanta Biologicals, Flowery Branch GA) and 1% vol/vol of Penicillin-Streptomycin (P/S, Gibco). CCL-81 cells were grown to ≈~90% confluence in 12-well tissue culture plates. Plasma samples were diluted in singlicate 1:1 (vol:vol) in Dulbecco’s Phosphate Buffered Saline (DPBS, no added Ca2+ or Mg2+) in Row A of 96 well tissue culture plates to a final volume of 150μL. Using a multichannel pipette, plasma and diluent were mixed and then 15μL were moved into 135μL of DPBS in Row B of the plate to represent the 20-fold dilution. Using a multichannel pipette, samples in Row B were mixed and then 15μL were moved into 135μL of DPBS in Row C of the plate to represent the 200-fold dilution. To serve as a positive control a six-step serial dilution series (from undiluted to 10^-5^) of YFV 17DD stock was conducted using DPBS. Subsequently, 100μL of these virus-serum mixtures were added to wells in the 12-well plates containing monolayers of CCL-81 cells. After a one-hour incubation at 37°C with 5% CO_2_ and agitation every 15 minutes, an overlay consisting of 0.8% methylcelluose in 1X MEM supplemented with 2% heat inactivated FBS and 1% P/S was added to each well. Plates were incubated for 96 hours at 37°C with 5% CO_2_, at which point the overlay was removed, plates were washed twice with 1X DPBS, and fixed for 30 minutes using methanol:acetone (1:1, vol:vol). Following fixation, methanol:acetone was removed and plates were allowed to air-dry overnight. Plates were then washed 3X for 15 minutes per wash with non-sterile 1X phosphate buffered saline on an orbital rocker, blocked for 15 minutes in a buffer of 3% FBS in DPBS, then incubated with rocking with a 1:2000 dilution of YF (Asibi) hyperimmune mouse ascitic fluid (HMAF) in 3% FBS in DBPS overnight. Plates were washed 3X for 15 minutes per wash with non-sterile 1X phosphate buffered saline on an orbital rocker, then incubated for 1 hour with rocking in a 1:2000 dilution of Affinity Purified Peroxidase Labeled, Goat-anti-mouse IgG (H+L) in 3% FBS in DPBS. Plates were washed 3X for 15 minutes per wash with non-sterile 1X phosphate buffered saline on an orbital rocker then developed using the AEC Peroxidase substrate kit according to manufacturer instructions. After development and drying, the plates were enumerated according to the following formula: [(# of foci in well)/(reciprocal of dilution factor)]/(inoculation volume in mL). The lowest number of plaques detected in multiple wells was 1 pfu. The LLOD for this assay was 20 pfu/mL.

**Neutralizing Antibody.**

Plaque-reduction neutralization tests (PRNT) were performed in monolayer cultures of Vero cells. Serum samples were heat-inactivated (56°C for 1 hour). 15μL of serum was mixed with 135μL of DMEM supplemented with 2% heat inactivated fetal bovine serum (FBS) and 1% P/S to generate a 1:10 dilution. A 5 step 2-fold serial dilution series was conducted 1:320. For YFV 17DD assays, YF (Asibi) HMAF was diluted alongside serum samples to serve as a positive control. Subsequently, challenge virus YFV 17DD was diluted to a known titer (800 IU/mL) and 75μL were mixed with 75μL of serum dilutions. These as well as 2-fold dilution series of virus only (controls) were allowed to incubate for 1 hour at 37°C 5% CO_2_. Subsequently, 100μL of virus/serum mixture or virus only control were added sequentially to each well of the 12-well plates containing monolayers of CCL-81 Vero cells. After a one-hour incubation at 37°C with 5% CO_2_ and agitation every 15 minutes, an overlay consisting of 0.8% methylcelluose in 1X MEM supplemented with 2% heat inactivated FBS and 1% P/S was added to each well. Fixation and staining were conducted as described above for YFV plaque assays. After development and drying, plates were enumerated. Control (virus only) plates were utilized to determine the number of plaques in a 1:1 diluted control well via averaging of 3-5 of such wells. The 50%, 60%, 80% and 90% plaque reduction titer values were determined from this average. If no neutralization was observed over the range of the assay, the PRNT value was reported as <1:20.

The PRNT for NiV antibodies is performed by UTMB in the BSL4 laboratories as described for YF but using NiV Malaysia as the challenge virus.
